# Mitochondrial dysfunction and its association with age-related disorders

**DOI:** 10.3389/fphys.2024.1384966

**Published:** 2024-07-02

**Authors:** Indumathi Somasundaram, Samatha M. Jain, Marcel Blot-Chabaud, Surajit Pathak, Antara Banerjee, Sonali Rawat, Neeta Raj Sharma, Asim K. Duttaroy

**Affiliations:** ^1^ Biotechnology Engineering, Kolhapur Institute of Technology’s College of Engineering, Kolhapur, India; ^2^ Department of Biotechnology, Faculty of Allied Health Sciences, Chettinad Hospital and Research Institute, Chettinad Academy of Research and Education, Chennai, India; ^3^ C2VN, Aix-Marseille University, Marseille, France; ^4^ Stem Cell Facility, DBT-Centre of Excellence for Stem Cell Research, All India Institute of Medical Sciences, New Delhi, India; ^5^ School of Bioengineering and Biosciences, Lovely Professional University, Phagwara, India; ^6^ Department of Nutrition, Faculty of Medicine, Institute of Basic Medical Sciences, University of Oslo, Oslo, Norway

**Keywords:** mitochondrial dysfunction, mitochondrial DNA, ROS production, chromosomal aberrations, mitophagy

## Abstract

Aging is a complex process that features a functional decline in many organelles. Various factors influence the aging process, such as chromosomal abnormalities, epigenetic changes, telomere shortening, oxidative stress, and mitochondrial dysfunction. Mitochondrial dysfunction significantly impacts aging because mitochondria regulate cellular energy, oxidative balance, and calcium levels. Mitochondrial integrity is maintained by mitophagy, which helps maintain cellular homeostasis, prevents ROS production, and protects against mtDNA damage. However, increased calcium uptake and oxidative stress can disrupt mitochondrial membrane potential and permeability, leading to the apoptotic cascade. This disruption causes increased production of free radicals, leading to oxidative modification and accumulation of mitochondrial DNA mutations, which contribute to cellular dysfunction and aging. Mitochondrial dysfunction, resulting from structural and functional changes, is linked to age-related degenerative diseases. This review focuses on mitochondrial dysfunction, its implications in aging and age-related disorders, and potential anti-aging strategies through targeting mitochondrial dysfunction.

## Graphical Abstract

**Figure:**
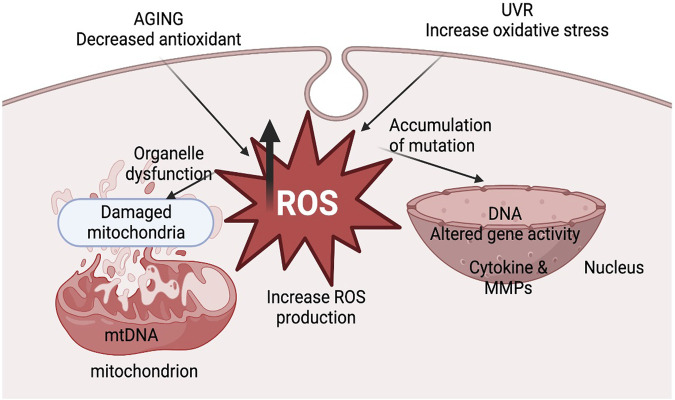
The figure shows the effect of mitochondrial dysfunction and mitophagy on stem cells leading to aging.

## Introduction

According to the United Nations (UN) in 2020 there were around 727 million people aged 65 years or over, and in the future, the number of aged people will increase compared to the younger population (United Nations Department of Economic and Social Affairs, P. D., 2020). This suggests a need to understand the aging process to improve individuals’ overall life expectancy and health. In humans, aging generally begins after thirty (Dziechciaz M and Filip, R., 2014) as a result of aggregation of physical, physiological, psychological, and social changes leading to an overall decline in physical and mental wellbeing and a reduction in mobility. Aging is a complex, multifactorial, and long-term process affecting individuals at various levels, including molecular, cellular, tissue, organ, and system (Cohen, 2018). Aging is associated with the onset of various age-related diseases and conditions, including cardiovascular diseases (Kizhakekuttu and Widlansky, 2010) such as atherosclerosis, hypertension, and heart problems, along with other diseases such as diabetes mellitus, arthritis, cataracts, hearing loss, weakened immune response, cancer, onset of neurodegenerative diseases such as Alzheimer’s disease and Parkinson’s disease in the later stages of life (Liu, 2014; Balmik and Chinnathambi, 2018; Li et al., 2021). Studies addressing aging in humans and animal models identified various cellular processes and events associated with aging and age-associated diseases (Mitchell, S.J, et al., 2015; Casajus Pelegay et al., 2019). López-Otín et al. (2013) proposed nine hallmarks of aging: genomic instability, telomere attrition, epigenetic alterations, loss of proteostasis, deregulated nutrient sensing, cellular senescence, stem cell exhaustion, altered intracellular communication, and mitochondrial dysfunction (Liu et al., 2002). The crosstalk between these hallmarks of aging has been identified, which has shed more light on the molecular mechanism of the aging process. Among these hallmarks of aging, mitochondrial dysfunction has been comprehensively studied as its role in aging (Sun et al., 2016; Kauppila et al., 2017; Zhang et al., 2018; Bar-ziv et al., 2020). Moreover, the crosstalk of mitochondrial dysfunction with other hallmarks of aging suggests that mitochondrial dysfunction actively influences the aging process by contributing to other hallmarks of aging (Van der Rijt et al., 2020).

Mitochondria are unique double-membrane organelles that came into existence due to the engulfment of alpha-proteobacterium by a eukaryotic progenitor cell in an endosymbiosis process, demonstrating evolutionary importance in the advancement of eukaryotic life (Lane and Martin, 2010). Mitochondria, apart from the nucleus, comprises its genome, metabolome, transcriptome, and proteome (Cloonan et al., 2020). Mitochondria have been considered a cellular powerhouse, responsible for approximately 95% of cellular ATP production. They are responsible for significantly contributing to the maintenance of cellular homeostasis by contributing to metabolic processes such as the tricarboxylic acid cycle (TCA) and oxidative phosphorylation (OXPHOS) (Tzameli, 2012; Birsoy et al., 2015). Apart from regulating cellular energetics, mitochondria also play an essential role in intracellular calcium signaling thermogenesis, apoptosis, generation of reactive oxygen species (ROS), and regulation of oxidative stress response (Kowaltowski, 2000). Any defect or deficit in mitochondrial number and function might be responsible for cellular damage. Mitochondrial dysfunction has been reported to be associated with aging and almost all chronic aging-associated diseases (Nicolson, 2014; Nehlin, 2023) through reduced ATP production, alteration in the regulation of apoptosis, increased ROS production, and defective calcium signaling (Norat et al., 2020). Accumulation of mutations in mitochondrial DNA (mtDNA) is the primary cause of mitochondrial anomalies, further contributing to aging and associated diseases (Kujoth et al., 2007; Paraskevaidi et al., 2017). Here, we provide a detailed description of mitochondrial dysfunction, its implications in the aging process, the onset of aging-associated diseases, and potential therapeutic interventions targeting mitochondrial dysfunction to develop an effective strategy for treating age-related diseases.

## Mutations in mitochondrial DNA (mtDNA) and aging

The human mitochondrial genome comprises small double-stranded circular DNA spanning 16,569 bp, which contains 37 genes coding for 13 polypeptides, 2 rRNAs, and 22 tRNAs. All these 13 polypeptides encoded by mtDNA are active components of the oxidative phosphorylation system (Taanman, 1999). Mitochondria contain several copies of mtDNA, and its replication is a cell cycle-independent and relatively dynamic process (Bogenhagen, and Clayton, 1977). Replication of mtDNA is regulated by several specific mitochondrial proteins such as mitochondrial polymerase gamma A (PolγA), TWINKLE and the mitochondrial single-strand binding protein (mtSSB) (Bogenhagen, and Clayton, 1977). mtDNA has been organized into nucleoids, aggregates of one or more mtDNA copies linked to proteins that bind to mitochondrial DNA, like mitochondrial transcription factor (TFAM) (Bogenhagen, 2012). The Association of mtDNA with DNA binding proteins protects the DNA against ROS and reactive nitrogen species (RNS) produced during mitochondrial metabolism (Bogenhagen, 2012). mtDNA has up to a 15-fold higher mutation rate and a less efficient DNA repair mechanism compared to nuclear DNA (Short et al., 2005). Accumulation of mutations in mtDNA beyond a critical threshold may lead to significant adverse effects in mitochondrial functioning and mutations in components of OXPHOS complexes, resulting in mitochondrial dysfunction and increased ROS production (Wallace, 2010). According to a few studies, age-associated increases in point and deletion mutations in mtDNA have been observed in various human tissues, including the brain, heart, colon and skeletal muscle (Bua et al., 2006; Marzetti and Leeuwenburgh, 2006; Kennedy et al., 2013; Greaves et al., 2014). However, the role of these mutations in the aging phenotype and whether they are the cause or effect of aging is still unclear. A study (Bratic et al., 2015) in the mtDNA mutator mouse model gave more insights into the accumulation of mutations in mtDNA and its implications in aging phenotypes. This model was developed by homozygous knock-in of PolγA lacking exonuclease activity (Bratic et al., 2015). Another study (Edgar and Trifunovic, 200) suggested that an increase in the frequency of mutations in mtDNA resulted in a shortened lifespan and premature onset of aging phenotype, including reduced fertility, anemia, osteoporosis, hair loss, the curvature of the spine, reduced body weight and premature death in this mouse (Edgar and Trifunovic, 2009).

The accumulation of mutations in mtDNA and its implications in the aging process. More research (Ross et al., 2013) using mice with a nuclear genome of wild type and mtDNA mutations inherited from a mother heterozygous for mutator allele (PolγAwt/mut) showed that low levels of germline-transmitted mtDNA mutations may have long-term effects, including early aging and a decrease in lifespan (Ross et al., 2013; Ross et al., 2014).

## Increased mitochondrial ROS production and aging

Mitochondria hosts the OXPHOS and ATP production with assistance from the electron transport chain located in the inner membrane of mitochondria. The electron transport chain located in the inner mitochondrial membrane consists of four protein complexes and is coupled with ATP synthase, an ATP-producing enzyme. ROS are considered to be unwanted and toxic by-products of the mitochondrial electron transport system (Chistiakov et al., 2014). ROS are extremely reactive chemical species that include superoxide anion (O_2_^−^), hydroxyl radical (OH), and hydrogen peroxide (H_2_O_2_). Because they tend to donate or acquire another electron in order to achieve stability, these radicals have a single unpaired electron, which contributes to their high reactivity. Hydrogen peroxide (H_2_O_2_), hypochlorous acid (HOCl), hypobromous acid (HOBr), ozone (O_3_), singlet oxygen (1O_2_), nitrous acid (HNO_2_), nitrosyl cation (NO^+^), nitroxyl anion (NO^−^), dinitrogen trioxide (N_2_O_3_), dinitrogen tetraoxide (N_2_O_4_), nitronium (nitryl) cation (NO_2_^+^), organic peroxides (ROOH), aldehydes (HCOR), and peroxynitrite (ONOOH) are the non-radical species. Even while these non-radical species may readily trigger free radical reactions in organisms, they are not free radicals themselves.

The mitochondrial electron transport system produces almost 90% of total cellular ROS, making it a major ROS generator in cells (Bratic and Trifunovic, 2010). ROS are highly reactive molecules that can lead to oxidative deterioration of molecules like proteins, lipids, and DNA. ROS leads to various DNA lesions, including oxidized DNA bases and DNA strand breaks (Cui et al., 2012). Besides its harmful effects in inducing oxidative stress in cells, ROS also acts as a signaling molecule that influences various cellular pathways and enhances immunologic defence against pathogens (Yang and Lian, 2020). Many studies (Liu et al., 2002; Bowling and Beal, 1995) focused on aging demonstrate that ROS is one of the major mediators of age-related cellular damage and the pathogenesis of some age-associated neurodegenerative diseases. Furthermore, many theories explaining the aging process, including the free radical theory and the mitochondrial theory of aging, are based on the harmful effects of ROS and associated cellular damage (Chistiakov et al., 2014; Yan, 2014; Indo et al., 2015). Although elevated levels of ROS typically lead to cellular harm and accelerate aging, lower amounts might enhance overall defence mechanisms by triggering an adaptive reaction. This phenomenon has been termed mitochondrial hormesis. (Ristow and Schmeisser, 2014).

**FIGURE 1:**
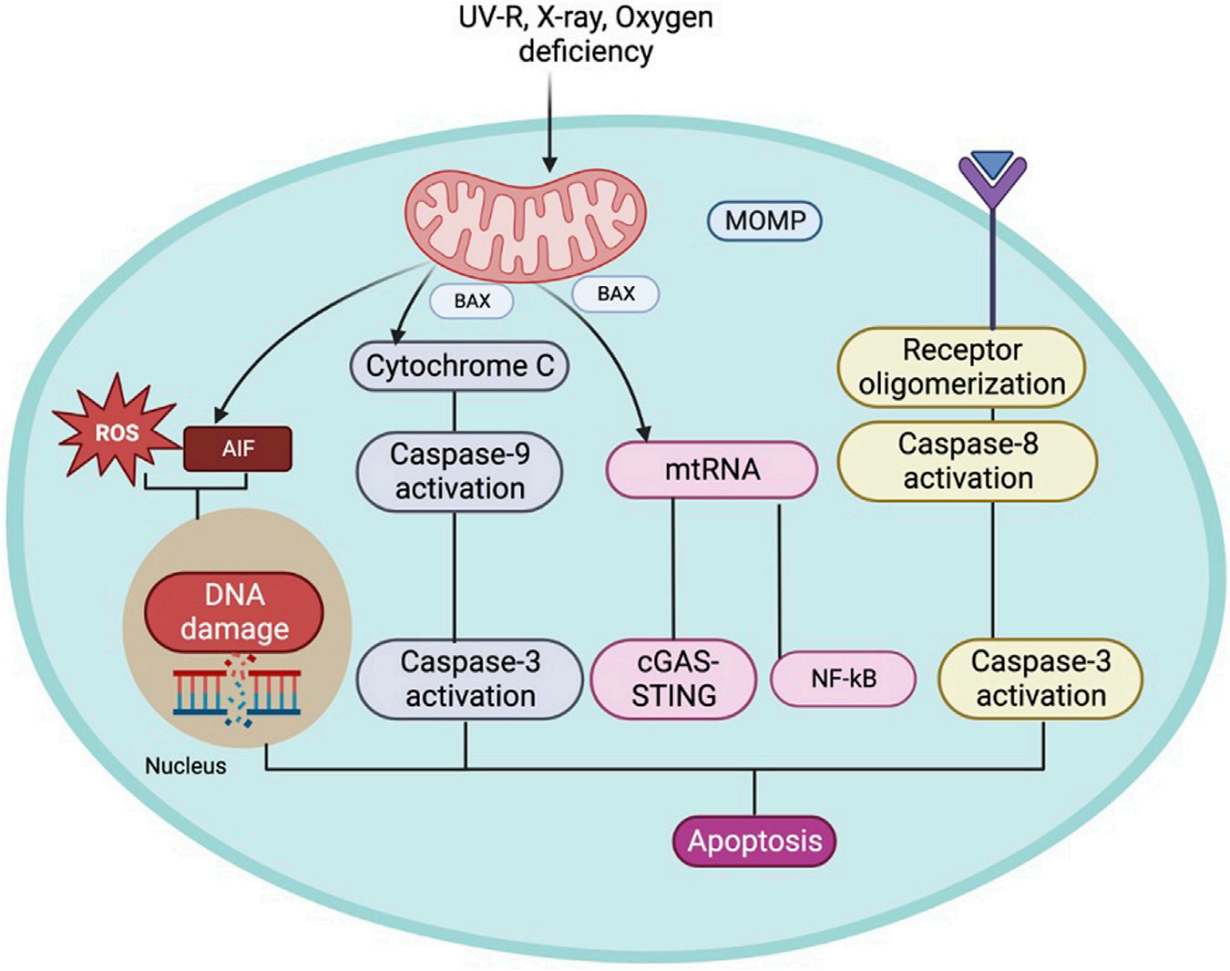
The illustration represents mitochondrial dysfunction and ROS production in aging.

The cumulative damage to mitochondria and accumulation of mutations in mtDNA caused by ROS leads to deterioration in mitochondrial functions due to alteration in the expression of components of the electron transport chain. ROS-mediated damage in OXPHOS machinery alters the enzymatic activity of mitochondrial respiratory enzymes and reduces mitochondrial membrane potential, leading to compromised ATP production (Cedikova et al., 2016). There are two major sites of ROS production in the electron transport chain: Complex I and Complex III. Complex I is the major site affected by increased ROS production. Seven complex I components are encoded by mtDNA, which gets frequently mutated due to ROS, ultimately contributing to the aging process (Indo et al., 2015; Cedikova et al., 2016). According to one report by Nissanka and Moraes (2018), the activity of complex I is significantly hampered due to aging in rat brain and liver along with human platelets, leading to enhanced ROS generation (Nissanka and Moraes, 2018). This suggests that there might be a positive correlation between a defect in complex I activity and ROS generation and *vice versa* and their overall impact on the aging process. According to the free radical theory of aging, free radical production *via* mitochondria, oxidative damage of tissues, aging, and aging-related diseases are interlinked, eventually affecting an individual’s physiology and metabolism. These include -the absence of specific growth factors, hormones, DNA damage, chemotherapeutic agents, serum starvation, UV radiation, toxins, certain conditions like hyperthermia, hypoxia, viral infections, free radicals affecting mitochondrial membrane potential, increased levels of Ca+2, formation of reactive oxygen and nitrogen species and decline in the redox status as (i.e., glutathione, ATP, NADH) (Elmore, 2007; Pollack and Leeuwenburgh, 2001). Transgenic mice’s lifespans have been examined in another investigation to determine the impact of overexpressing antioxidant enzymes. The overexpression of either copper zinc superoxide dismutase (CuZnSOD) and catalase or CuZnSOD and manganese superoxide dismutase (MnSOD) may occur. Superoxide and hydrogen peroxide in the cytosolic and mitochondrial compartments are known to be scavenged by the overexpression of these important antioxidant enzymes. Therefore, the life span of mice is not increased by the overexpression of antioxidant enzymes (Pérez et al., 2009). This occurs because the increased levels of ROS can cause damage to mitochondrial DNA, as shown in Figure 1, leading to a decline in mitochondrial function. This decline in function, in turn, leads to increased oxidative stress that triggers a signaling cascade that contributes to skin structure and photoaging changes. Furthermore, through certain signaling genes like NF-kβ and AP-1, ROS can increase the expression of proinflammatory cytokines and induce inflammation. ROS can also directly damage the skin by increasing the expression of MMP (Benz and Yau, 2008; Rizwan et al., 2014). ROS-induced DNA mutations, lipid peroxidation, and protein oxidation are all implicated in skin aging and photoaging development (Rinnerthaler et al., 2015).

## Mitochondrial apoptotic pathway in aging

**FIGURE 2:**
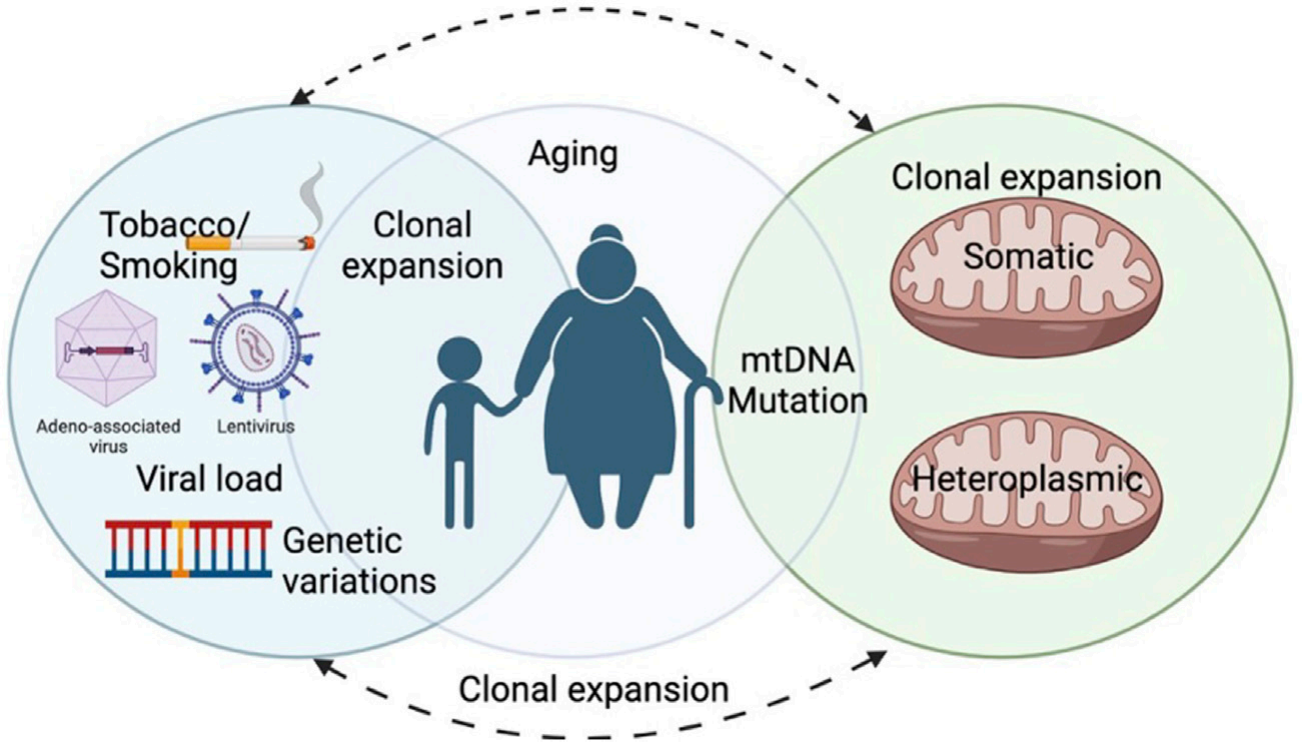
The figure represents the mitochondrial role leading to cell apoptosis and its association in aging.

Apoptosis is a homeostatic mechanism essential in controlling cell death, maintaining the average cell turnover, embryonic development, effective immune system functioning, maintaining cellular and tissue homeostasis, maintaining cell growth, differentiation, and tissue repair (Greenhalgh, 1998; Alberts et al., 2002; Mondello and Scovassi, 2010; Tower, 2015). This mechanism is executed without detrimental effects on the neighbouring cells as the process does not release cellular components into the surrounding tissue environment (Alberts et al., 2002; Kurosaka et al., 2003). The mitochondrial-mediated apoptosis pathway is initiated due to various death signals (Ravindran et al., 2011). The Bcl-2 family proteins are also critical in regulating the apoptosis progression by influencing the mitochondrial membrane permeability and Ca+2 influx, releasing cytochrome c, blocking electron transfer between complexes III and IV of the electron transport system, and preventing ATP generation. Cytochrome C is released from the mitochondria to the cytosol and activates Apaf-1, forming an “apoptosome” complex along with caspase-9, following cleavage and activation of the effector protease, caspase-3, leading to apoptosis. The pro-apoptotic and anti-apoptotic proteins trigger apoptosis or terminate the apoptosis progression by influencing the mitochondrial membrane permeability transition pores (mPTP), which is shown in (Figure 2) (Pollack and Leeuwenburgh, 2001; Eberle and Hossini, 2008). These observations (Srivastava, S., 2017) suggest that the mitochondrial apoptosis pathway plays a pivotal role in the aging process and pathology associated with age-related diseases. Additionally, mtRNA can cause apoptosis independently through the NF-κB and cGAS-STING pathways (Decout et al., 2021). If the permeability of the mitochondria increases, they will release apoptosis-inducing factors (AIF) which, when released into the cytoplasm or nucleus, can damage DNA and ROS, triggering apoptosis (Camplejohn et al., 2003; Sevrioukova, 2011).

Age-associated alterations in Mitochondrial Dynamics and the role of mitokines in aging: The mitochondrial network is regulated by the complex fission, fusion, and distribution events, which are essential for normal mitochondrial functions (Roy et al., 2015). A fine balance between mitochondrial fusion and fission events is maintained to optimize mitochondrial function. Mitochondrial fusion events are responsible for fusing mitochondrial contents to reduce the impact of damaged mtDNA and proteins (Silva Ramos et al., 2019). On the other hand, mitochondrial fission events are responsible for removing damaged mitochondrial contents by selective mitochondrial autophagy (Rubinsztein et al., 2011; Papackova, and Cahova, 2014). Mitochondrial biogenesis is a complex process regulated by the coordination of nuclear and mtDNA and triggered by factors such as hypoxia, caloric restriction, fasting, cold, physical exercise and hormonal stimulation. PGC-1α, PGC-1β, and PGC-1 are the family of PGC-1 proteins that activate mtDNA transcription; PGC-1α is regarded as the primary regulator of mitochondrial biogenesis.

Further, the nuclear transcription factors nuclear respiratory factor-1 (NRF-1), NRF-2, and estrogen-related receptor-α (ERR-α) are stimulated, and TFAM, the last effector of mtDNA transcription and replication, is expressed more frequently. The pathway is started by PGC-1α activation (either by phosphorylation or deacetylation). NRFs and ERRα modulate the expression of mitochondrial transcriptional factors and regulate the expression of mitochondrial respiratory subunits to initiate the process of biogenesis (Scarpulla, 2008; Zhu et al., 2013; Wang J. et al., 2020). There is an intricate balance between mitochondrial biogenesis and mitophagy, and defects in these mitochondrial dynamics may lead to alterations in mitochondrial and cellular functions. Altered mitochondrial dynamics have implications in the modulation of cellular processes that might be associated with aging (Terman et al., 2010).

Impaired mitochondrial dynamics have been implicated in age-associated changes due to functional and structural changes in mitochondria (Cedikova et al., 2016). According to a few studies, age-associated structural and functional mitochondrial defects have been observed in skeletal muscles, liver, kidney, heart, brain, diaphragm muscles, bone and fibroblasts (Yen et al., 1989; Shigenaga et al., 1994; Crane et al., 2010; Terman et al., 2010; Mahmoud and Hegazy, 2016; Marycz et al., 2016; Shum et al., 2016; Palomera-Avalos et al., 2017). In another study, a decline in mitochondrial density was observed in skeletal muscles with age progression (Crane et al., 2010). Mitochondrial fusion is mainly facilitated by two mitofusins, Mfn1 and Mfn2. These two mitofusins are on the outer membrane of mitochondria and play an important function in mitochondrial membrane fusion (koshiba, et al., 2004). According to one study, loss of Mfn1 and Mfn2 in mice increases mtDNA mutations in skeletal muscles, suggesting that mitofusins are important for mitochondrial integrity (Chen et al., 2010).

Moreover, decreased expression of Mfn2 protein and mRNA in the frontal cortex has been reported in patients with Alzheimer’s disease (Chen et al., 2010; Manczak et al., 2011). Age-associated defects in mitochondrial dynamics, along with impaired mitochondrial structure and functions, have been reported in various studies, indicating the importance of mitochondrial dynamics in health and aging.

Another important aspect of mitochondrial dynamics involves the cross-talk between mitochondria of distant cells under stress conditions through signalling molecules called mitokines. Mitokines include molecules expressed in the nucleus such as Fibroblast growth factor 21 (FGF21) and Growth Differentiation Factor-15 (GDF15) as well as mitochondrial derived peptides like humanin (HN) (Burtscher et al., 2023). Mitokines are essential factors to ensure cellular vitality and mitochondrial functions under stress conditions and other external stimulus in the form of exercise and diet (Burtscher J, et al., 2023). Mitochondria modifies its functions through metabolic reprogramming and epigenetic remodelling upon oxidative stress, impaired, ETC or proteotoxic stress generated by unfolded proteins (Mottis et al., 2019; Boos et al., 2020; Zhou et al., 2022). The dysregulation in mitokines signalling in the form of mitokines resistance or mitokines synthesis results in development of age-related decline in physiological functions and manifestation of metabolic, cardiovascular or neurological diseases. The plasma concentrations of FGF21 and GDF15 have increased with aging in humans and have been correlated to cardiovascular, metabolic and neurological diseases (Youm et al., 2016; Conte et al., 2019). Furthermore, mitokines such as HN and mitochondrial ORF of the 12S rRNA type-c (MOTS-c) play a protective role against cellular stress and inflammation. Additionally, HN has been shown to enhance insulin sensitivity and increase glucose transported 4 (GLUT4) expression in rodent model of diabetes (Lee et al., 2015; Wu et al., 2022). The levels of circulating HN are upregulated in aging individuals, suggesting its protective role against age-associated metabolic complications (Conte et al., 2019). Improved balance in mitokines level has been reported in individuals performing regular moderate to intensive exercise leading to improved cellular function and longevity (Taniguchi et al., 2016; Zhang et al., 2019). The role of mitokines in aging need to be characterized through intensive research to develop mitokines based therapeutic strategies against aging.

## Mitophagy and aging

Autophagy is the controlled process associated with recycling components of the cell by delivering them to the lysosome for degradation under special circumstances such as nutrient starvation, dysfunction of particular cellular components, removal of cellular protein aggregates, control of cellular biomass, and elimination of intracellular pathogens (Ohsumi, 2014). Autophagy has been studied extensively concerning the aging process as a decrease in autophagy has been observed with aging (Rubinsztein et al., 2011; Madeo et al., 2010). Autophagy of mitochondria is known as mitophagy, which occurs during nutrient starvation, selective removal of mitochondria during differentiation of cells such as sperms, ocular lens cells, and red blood cells, as well as for the removal of dysfunctional mitochondria (Sato and Sato, 2013; Costello et al., 2013; Mortensen et al., 2010; Lemasters, 2005). Mitophagy has been associated with several conditions such as oxidative stress, hypoxia, defect in the electron transport chain, accumulation of protein aggregates, and iron starvation. Impairment in the mitophagy process has been associated with various pathological conditions, including aging and aging-associated diseases such as neurodegenerative diseases, cardiovascular diseases, and cancer (Chen et al., 2020; Evangelou et al., 2023).

The major regulator of mitophagy of dysfunctional mitochondria is PTEN-induced putative kinase 1 (PINK-1), and this process is also known as PINK1-mediated mitochondrial quality control (Wall et al., 2019). A defect in membrane potential dissipation prevents PINK-1 degradation followed by its autophosphorylation, which activates E3-ubiquitin ligase Parkin. Parkin promotes the ubiquitination of mitochondrial membrane proteins, further phosphorylated by PINK1. These phosphorylated polyubiquitination chains are recognized by adaptor proteins of core autophagy machinery such as p62 and OPTN, which further interacts with LC3 and initiates autophagosome formation around dysfunctional mitochondria, which is further fused with lysosome, leading to autolysosome formation and ultimately undergoes degradation (Palikaras et al., 2018). A widely studied pathway for mitophagy involves the PINK1/Parkin mechanism, wherein PINK1 recruits Parkin to dysfunctional mitochondria, triggering their degradation. However, aside from PINK1/Parkin-mediated mitophagy, alternative pathways exist that operate independently. One such pathway is SLR-independent mitophagy, wherein damaged mitochondria are identified by specific receptors like NIX/BNIP3L and FUNDC1. These receptors then engage with the autophagy machinery to facilitate degradation. In contrast, SLR-independent mitophagy pathways utilize different mechanisms for recognizing and targeting dysfunctional mitochondria for degradation. These pathways represent diverse strategies within cells for maintaining mitochondrial quality control and cellular homeostasis (Ganley and Simonsen, 2022).

Various studies involving model organisms such as *Caenorhabditis elegans* and *Drosophila melanogaster* have demonstrated defects in mitophagy and its implications for the health and lifespan of the organism (Bakula and Scheibye-Knudsen, 2020). Moreover, a study in transgenic mice expressing the fluorescent mitophagy reporter mt-Keima suggested that with age, there was a decrease in the mitophagy in the hippocampus region of the mouse brain (Sun et al., 2015). Also, decreased mitophagy has been observed in mouse hearts, and its implications in heart aging have been documented (Hoshino et al., 2013). Further association between defects in mitophagy and cardiovascular diseases has been reported, suggesting the role of decreased mitophagy in aging and age-associated diseases (Bravo-San Pedro et al., 2017). Decreased mitophagy has been reported to contribute to the pathogenesis of several age-associated disorders such as Parkinson’s disease, Alzheimer’s disease, cardiomyopathies, and cancer (Bernardini et al., 2017; Fivenson et al., 2017; Levine and Kroemer, 2019). The exact mechanism underlying decreased mitophagy in the aging process and age-associated diseases is still unclear, and more comprehensive studies are required to delineate detailed mechanisms.

Moreover, several researchers have attempted to target mitophagy as an effective strategy for developing anti-aging therapeutics (Bakula and Scheibye-Knudsen, 2020). Further analysis reveals that in the mito-QC reporter mouse, mitophagy in multiple organs appears to either increase or remain unchanged in older mice compared to younger ones. Transcriptomic analysis reveals a significant upregulation of the type I interferon response in the retina of older mice, which is associated with elevated levels of cytosolic mtDNA and activation of the cGAS/STING pathway (Boya, 2024; Jiménez-Loygorri et al., 2024).

## Association of mitochondrial dysfunction with other hallmarks of aging

Increased mitochondrial ROS production in aging cells contributes to another hallmark of aging apart from genomic instability and telomere attrition: epigenetic alterations. Increased levels of cellular ROS affect the DNA methylation status, contributing to age-associated modifications in epigenetic signatures (Liu L et al., 2002; Kietzmann et al., 2017). Increased ROS production due to age-associated mitochondrial dysfunction leads to 8-hydroxylamine (8-OHdG) DNA lesions, further inhibiting DNA methylation. These 8-OHdG lesions caused by increased ROS production are the major reason behind altered DNA methylation patterns observed during aging (Turk et al., 1995).

**FIGURE 3:**
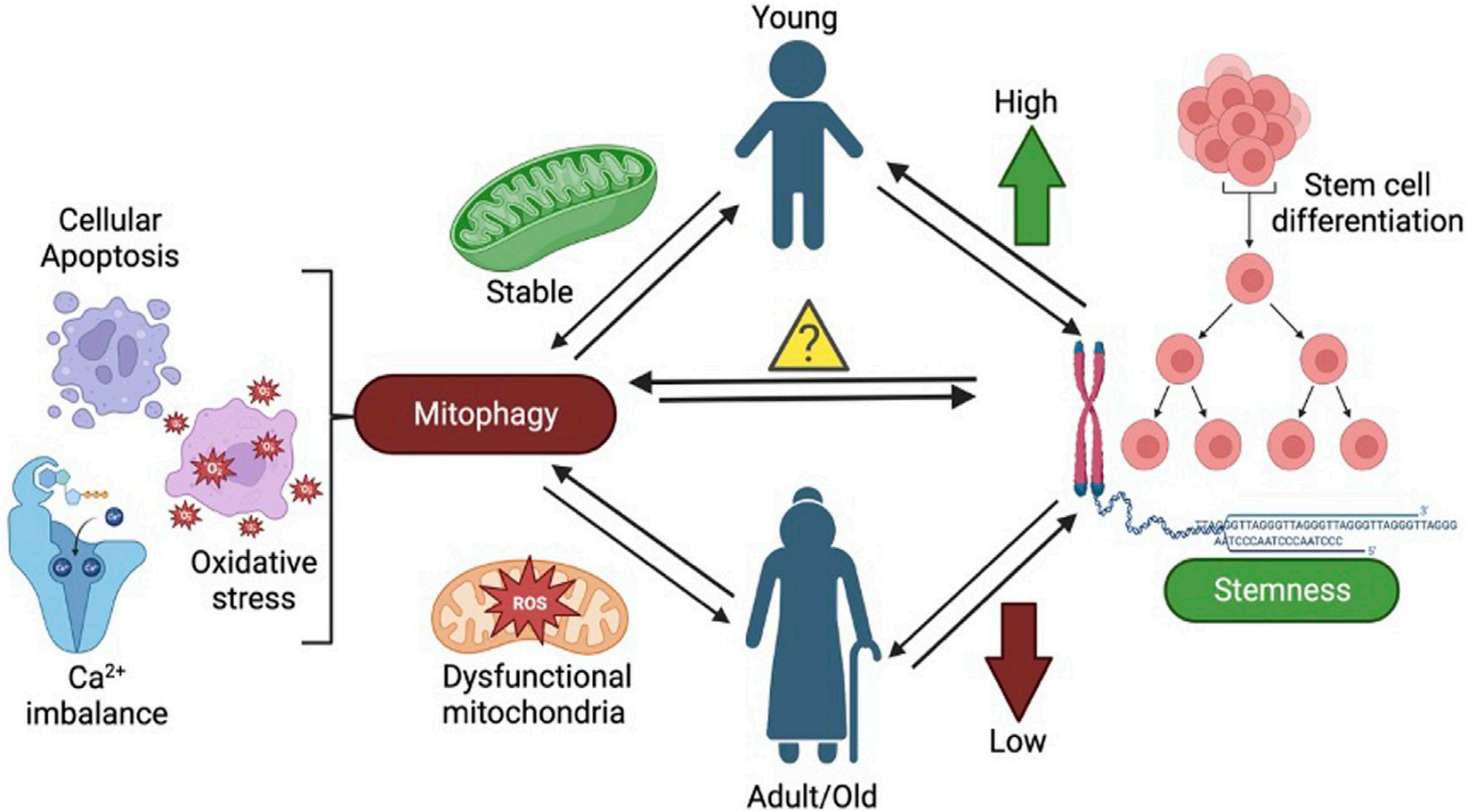
The representative illustration shows the Mutational burden and heteroplasmy in mitochondrial DNA that is linked to biological age, tobacco, and Viral infections.

Proper mitochondrial and cytosolic proteostasis is important for healthy aging and longevity (Molenaars et al., 2020). mTOR is the key molecule of the nutrient-sensing pathway. It is a key regulator of mitochondrial activity, biogenesis, and dynamics (Morita et al., 2013; Morita et al., 2017). mTOR is an important serine/threonine kinase involved in cellular pathways regulating growth and proliferation. By stimulating the synthesis of mitochondria-related proteins encoded in the nucleus, mTOR regulates the energy generation of the mitochondria and the energy consumption of the mRNA translation machinery. Research shows that by promoting the synthesis of mitochondrial transcription factor A (TFAM), mitochondrial ribosomal proteins, and elements of complexes I and V, mTOR regulates mitochondrial activities. By inducing the synthesis of TFAM, mitochondrial ribosomal proteins, and elements of complexes I and V, mTOR regulates the activities of mitochondria (Morita. M., 2015). Furthermore, the nutrient-sensing mechanism/mammalian target of rapamycin complex 1 (mTORC1) plays a role in mitochondrial fission and apoptosis by stimulating the translation of mitochondrial fission process 1 (MTFP1) (Morita et al., 2015). The study (Zorov et al., 2014) highlights that both genetic interventions and pharmacological treatments targeting reactive oxygen species (ROS) resulting from dysfunctional mitochondria have demonstrated the normalization of several disease-related traits across different species. Additionally, dietary caloric restriction (CR) has proven effective and has recently been shown to decelerate the aging process in humans. Conversely, genetic modifications aimed at altering mitochondrial dynamics have yielded conflicting outcomes, potentially due to variations specific to cells, tissues, and the particular neurodegenerative diseases under investigation Figure 3 (Scott et al., 2019).

Another aspect of aging affected by mitochondrial dysfunction is cellular senescence. Oxidative stress generated by a defect in mitochondrial components induces cellular senescence (Moiseeva et al., 2009). Cells become senescent when they enter a non-proliferative state while remaining active metabolically. High levels of ROS characterize the senescent state as a result of mitochondrial deregulation. Conversely, mitochondrial deregulation can drive cellular senescence (Yoon et al., 2003; Yoon et al., 2006; Byun et al., 2012). Abrupted energy transduction pathway, imbalance in mitochondrial dynamics, increased AMPK activity, and deregulation of mitochondrial calcium homeostasis may lead to cell cycle arrest (Ziegler et al., 2015). Increased ROS production has been shown to increase the rate of telomere shortening in cells and trigger the onset of cellular senescence through sustained DNA damage response (Passos et al., 2007; Chen et al., 2015; Davalli et al., 2018). Apart from this, defect in mitochondrial dynamics is also associated with the induction of cellular senescence (Lee et al., 2007).

Nicotinamide adenine dinucleotide (NAD) is a metabolite well implicated in senescence. NAD + acts as an electron carrier during the process of oxidative phosphorylation. The ratio of NAD+/NADH becomes low in cells undergoing senescence due to mitochondrial dysfunction (van der Veer et al., 2007; Lee et al., 2012). The lower NAD+/NADH ratio has been suggested to arise due to reduced cytosolic malate dehydrogenase levels, which utilize NADH to convert oxaloacetate to malate during oxidative phosphorylation. Low NAD+/NADH ratio also triggers senescence through p53 by activating 5′AMP-activated protein kinase (AMPK) (Jones et al., 2005). The cellular senescence driven *via* various factors of mitochondrial dysfunction has been collectively termed mitochondrial dysfunction-associated senescence (MiDAS) (Chapman J. et al., 2019). Cells undergoing MiDAS are characterized by lower NAD+/NADH ratios, leading to cell growth arrest and IL-1-associated senescence-associated secretory phenotype (SASP) (Wiley et al., 2016). Along with other aging-related hallmarks, stem cell exhaustion is associated with increased ROS production (Jang and Sharkis, 2007). A study in mitochondrial mutator mice demonstrated neuronal and hematopoietic stem cell dysfunction (Trifunovic et al., 2004).

## Targeting mitochondrial dysfunction as an anti-aging therapeutic strategy

There is a need to improve health and increase the life expectancy of the aged population. As a result, current biomedical research has been heavily focused on aging to identify potential targets for prolonging human health and lifespan (Madreiter-Sokolowski et al., 2018). Interventions like caloric restrictions, exercise, and pharmacological therapies have been studied, which can effectively improve health and slow down phenotypes of the aging process (Madreiter-Sokolowski et al., 2018; Nilsson and Tarnopolsky, 2019). Studies show that mitochondrial dysfunction is one of the most important aspects of aging and age-related disorders (Morita et al., 2013; Wu et al., 2022). Current research in developing effective therapeutic interventions and strategies for treating aging phenotypes and aging-associated diseases is inclined to utilize mitochondrial dysfunction as an effective target (Nicolson and Ash, 2017; Balcázar et al., 2020; Rai et al., 2020; Zimmermann et al., 2021).

Behavioral interventions such as caloric restriction and exercise are widely studied for delaying the aging process. Their association with mitochondrial biogenesis and function has been established in various model organisms (Madreiter-Sokolowski et al., 2018). Caloric restriction has been known to improve mitochondrial respiration, reducing ROS production (Madreiter-Sokolowski et al., 2018). Besides this, caloric restriction promotes mitochondrial biogenesis by activating AMPK signaling, activating SIRT1 and TFAM, and increasing mtDNA replication and transcription (Civitarese et al., 2007). However, further studies are required to determine the time of caloric restrictions in different individuals for optimum output related to improving health and delay in the cellular aging process. Besides caloric restriction, exercise is another behavioral intervention that has been reported to improve health and lead to delays in the aging process by influencing mitochondrial functions (Nicolson and Ash, 2017; Balcázar et al., 2020). According to a study in mice, PGC-1α and mitochondrial SIRT3 get downregulated with aging, which was further normalized by exercise, leading to an improved ROS defense mechanism (Gioscia-Ryan et al., 2016). These observations suggest that a combination of caloric restriction and regular exercise might improve health and delay aging by improving overall mitochondrial functions.

Various pharmacological molecules targeting mitochondrial dysfunction have been identified, with potential anti-aging properties. Polyphenols such as resveratrol and green tea polyphenols have been reported to extend lifespan in various model organisms, including mice (Rai et al., 2020). Resveratrol improves mitochondrial function by SIRT1-dependent activation of AMPK in mouse models (Price et al., 2012). Apart from polyphenols, natural and synthetic compounds are reported to improve mitochondrial functions. Natural compounds such as Urolithin A, a gut metabolite of ellagic acid, have been widely studied for their anti-aging activity in model organisms such as *C. elegans* and rodents (Price et al., 2012).

**FIGURE 4:**
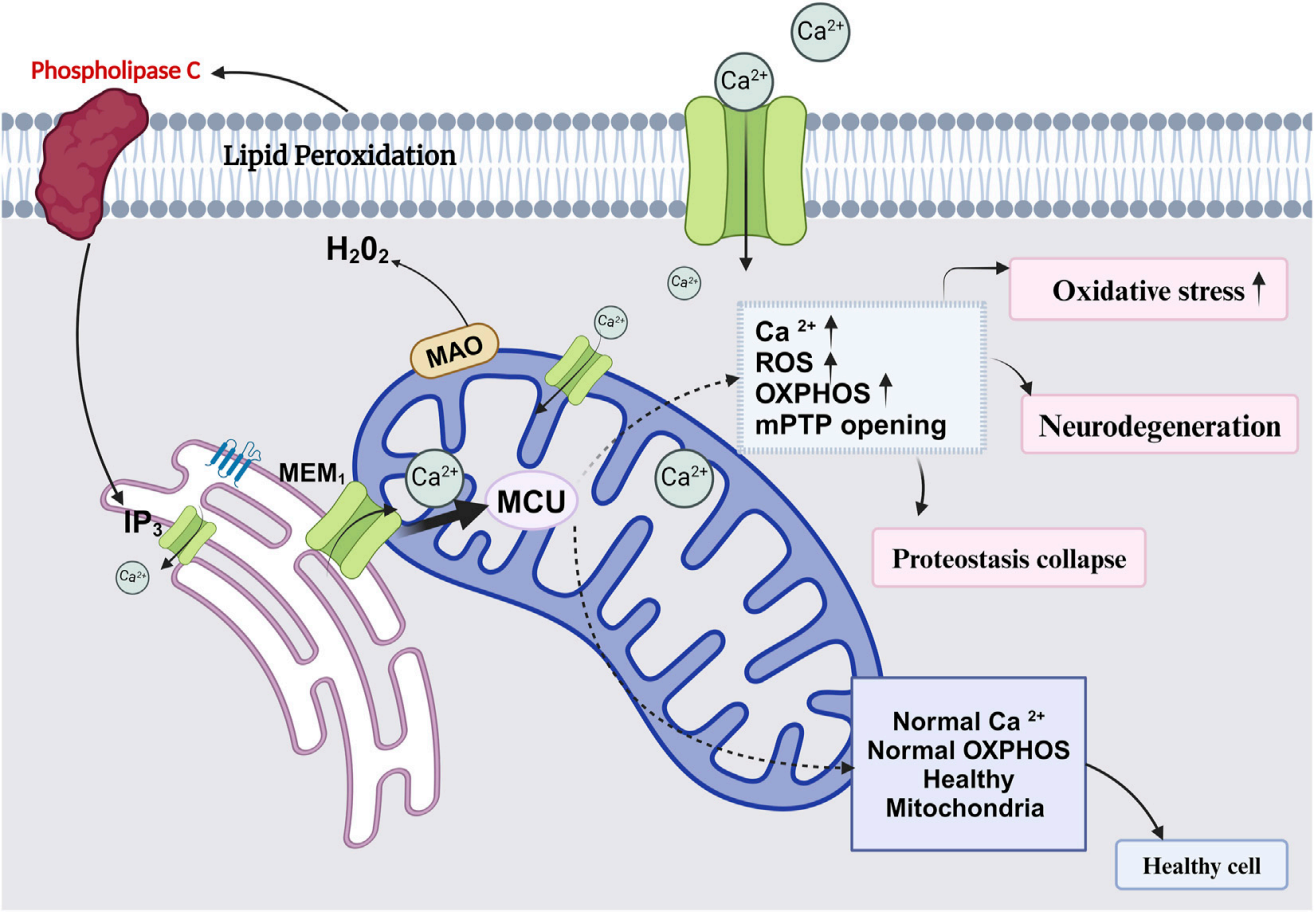
Diagram represents role of healthy and unhealthy mitochondria and its association in age associated diseases.

Moreover, Urolithin A has been reported to improve human mitochondrial and cellular health (Andreux et al., 2019). Apart from Urolithin A, the potential anti-aging activity of Actinonin and Polyamine spermidine *via* mitophagy induction has been reported (Bakula and Scheibye-Knudsen, 2020). N-3 fatty acids have also been studied to determine their anti-aging properties *via* improved mitochondrial ATP production and increased mitochondrial protein synthesis (Madreiter-Sokolowski et al., 2018). Melatonin has a multifaceted role as a free radical scavenger and a modulator of gene expression of antioxidant enzymes such as glutathione peroxidase and glutathione reductase, thus aiding in reducing oxidative damage (Ding et al., 2014; Kopustinskiene and Bernatoniene, 2021). It acts on mitochondrial uncoupling proteins (UCPs) and dissipates the proton gradient across the inner mitochondrial membrane (Agil et al., 2015; Tan, et al., 2016). The Chinese herb Scutellaria Baicalensis yields the flavonoid baicalein, which is a strong antioxidant and free radical scavenger while preventing the deposition of amyloid protein aggregates.

(Sonawane et al., 2019; Wang et al., 2021; Marković et al., 2011). More comprehensive studies are required to utilize these natural compounds as an effective anti-aging treatment to promote mitochondrial functions and reduce oxidative stress caused by mitochondrial dysfunction, as shown in Figure 4.

Apart from natural compounds, various synthetic compounds have been identified which demonstrate anti-aging activity by targeting mitochondrial functions. Randomized trials and population studies using natural antioxidants have produced poor results despite a substantial body of epidemiological and clinical data suggesting that antioxidant-rich diets lower blood pressure and cardiovascular risk (Kizhakekuttu and Widlansky, 2010). Apart from synthetic antioxidants, AMPK pathway activators, AICAR, and metformin have been identified as potential drugs for anti-aging therapy (Madreiter-Sokolowski et al., 2018). These AMPK pathway activators mimic the caloric restriction effects in the cells and lead to improved mitochondrial biogenesis, which further contributes to anti-aging properties (Toyama et al., 2016; Stancu, 2015). These studies suggest that targeting mitochondrial dysfunction and improving mitochondrial biogenesis could be a possible mode for developing anti-aging therapies. Furthermore, induction of mitophagy by behavioral interventions and treatment with natural and synthetic compounds might prove helpful in improving overall health and increasing life expectancy in the future.

Another approach to promote healthy aging in individuals has been directed towards cellular senescence. Therapeutics targeted against senescent cells are categorized into senolytics, i.e., killing senescent cells, and senomorphics/senostatics, i.e., targeted towards inhibition of SASP secretions (Atayik, and Çakatay, 2022; Nehlin, 2023; Van Deursen, 2014). Senolytic treatment has shown numerous beneficial effects in the form of improved genomic stability, alleviation of telomere attrition, mitochondrial dysfunction, and inflammatory response (Cho et al., 2023). Quercetin has been used as a senolyte which has been shown to induce AMPK-mediated apoptosis to eliminate senescent vascular smooth muscle cells and endothelial cells along with promoting mitophagy and NRF2-NFκB signaling (Wang L. et al., 2020; Jiang et al., 2020; Shao et al., 2022). Additionally, combined treatment of quercetin and Dasatinib is effective in improving overall cellular functions in aged hosts, decreasing the secretion of pro-inflammatory cytokines in human adipose tissues, and reducing senescent cell burden (Xu et al., 2018; Camell et al., 2021; Saccon et al., 2021). Fisetin is another flavonoid molecule present in various fruits and vegetables, known to exhibit senolytic effect (Khan et al., 2013). Additionally, fisetin facilitates the activation of Nfr2 in HUVECS, leading to hemeoxygenase one mediated antioxidant response (Yousefzadeh et al., 2018; Kwak et al., 2014). Piper longumine, an alkaloid in long peppers, inhibits platelet aggregation and thrombus formation (Chatterjee and Dutta, 1967; Iwashita et al., 2007). It has shown that the regulation of platelet-derived growth factor BB (PDGF-BB) mediated mitochondrial fission and mitochondrial protection in the case of hyperproliferation vascular phenomena (Salabei and Hill, 2013). Overall, the combination of various therapeutics interventions and senotherapeutic drugs can be effective in restoring mitochondrial functions and balancing mitochondrial dynamics, thereby targeting the mitochondria-associated aging phenotype to promote healthy aging.

Interestingly, TCA cycle metabolites can exhibit effects that oppose senescence. For example, α-ketoglutarate has demonstrated senomorphic properties by diminishing the SASP in senescent fibroblasts and extending the health span and lifespan in aged mice (Liao et al., 2021).

To begin, numerous senolytic medications presently under development exert their influence by focusing on anti-apoptotic proteins situated within mitochondria, which obstruct apoptosis driven by mitochondria. Senolytic drugs produce their senolytic impact by directing the anti-apoptotic B-cell lymphoma 2 (BCL-2) protein family within mitochondria (Basu, 2022; Martini, and Passos, 2023).

## Conclusion and future perspective

Mitochondria is a central organelle involved in the maintenance of cellular and physiological wellbeing, which contributes to cellular energetics and other important cellular processes such as cell signaling, calcium homeostasis, cell growth, and apoptosis. Systemic deterioration in mitochondrial components, functions, and dynamics has been associated with aging. Accumulation of mutations in mtDNA leads to increased production of defective mitochondrial proteins and increased ROS production. A further contribution of increased ROS production and activation of the mitochondrial apoptotic pathway in aged cells leads to systemic deterioration of various tissues, decreasing overall organ and tissue functions, including muscle, heart, liver, and brain function. Defective mitochondrial proteostasis has been associated with altered cellular functions in aging cells.

The decreased rate of mitophagy in aged cells further contributes to the accumulation of defective mitochondria and their effect on an organism’s cellular and overall health. The association of increased mitochondrial dysfunction with other hallmarks of aging makes it a possible target for developing therapies for treating aging phenotypes and associated disorders. Behavioral interventions and pharmacological compounds targeting mitochondrial dysfunction have increased hope in designing future therapeutic management of aging phenotypes and related diseases. Various therapeutic natural and synthetic molecules targeting mitochondrial functions have been studied for their implications on aging. There is still a need to identify the detailed molecular mechanism behind mitochondrial dysfunction, including the decrease in mitophagy in aging cells and their association with the overall aging process. Despite promising therapeutic interventions targeting mitochondrial dysfunction for treating pathologies associated with aging and age-related diseases, more comprehensive molecular and clinical studies are required to effectively utilize mitochondrial dysfunction as a target for anti-aging therapies.

## Abbreviations

ROS, Reactive oxygen species; mtRNA, Mitochondrial RNA; DNA, Deoxyribonucleic acid; UN, United Nations; TCA, tricarboxylic acid cycle; OXPHOS, Oxidative phosphorylation; ATP, adenosine triphosphate; mtSSB, Mitochondrial single strand binding protein; TFAM, Mitochondrial transcriptional factor A; RNS, Réactive nitrogen species; MMP, Matrix metalloproteinases; AP-1, Activator Protein-1; CTL, cytolytic T lymphocytes; mPTP, membrane permeability transition pores; PGC-1a, Peroxisome proliferator-activated receptor-gamma coactivator (PGC)-1 alpha; PINK-1, PTEN induced putative kinase 1; OPTN, Plk1 phosphorylates Optineurin; 8-OHDG, 8- hydrooxylamine; mTOR, mammalian target of rapamycin; MTFP1, Translation of mitochondrial fission process 1; TFAM, Mitochondrial transcription factor A; AMPK, AMP-activated protein kinase; SIRT1, sirtuin (silent mating type information regulation two homolog) 1; UCPs, Mitochondrial uncoupling proteins; DAMPs, Damage-associated molecular patterns; CNS, Central nervous systems.

## References

[B1] Agil A. El‐Hammadi M. Jiménez‐Aranda A. Tassi M. Abdo W. Fernández‐Vázquez G. (2015). Melatonin reduces hepatic mitochondrial dysfunction in diabetic obese rats. J. pineal Res. 59 (1), 70–79. 10.1111/jpi.12241 25904243

[B2] Alberts B. Johnson A. Lewis J. Raff M. Roberts K. Walter P. (2002). “Programmed cell death (apoptosis),” in Molecular biology of the cell. 4th edition (Germany: Garland Science).

[B3] Andreux P. A. Blanco-Bose W. Ryu D. Burdet F. Ibberson M. Aebischer P. (2019). The mitophagy activator urolithin A is safe and induces a molecular signature of improved mitochondrial and cellular health in humans. Nat. Metab. 1 (6), 595–603. 10.1038/s42255-019-0073-4 32694802

[B4] Atayik M. C. Çakatay U. (2022). Mitochondria-targeted senotherapeutic interventions. Biogerontology 23 (4), 401–423. 10.1007/s10522-022-09973-y 35781579

[B5] Bakula D. Scheibye-Knudsen M. (2020). MitophAging: mitophagy in aging and disease. Front. Cell. Dev. Biol. 8, 239. 10.3389/fcell.2020.00239 32373609 PMC7179682

[B6] Balcázar M. Cañizares S. Borja T. Pontón P. Bisiou S. Carabasse E. (2020). Bases for treating skin aging with artificial mitochondrial transfer/transplant (AMT/T). Front. Bioeng. Biotechnol. 8, 919. 10.3389/fbioe.2020.00919 32903493 PMC7438394

[B7] Balmik A. A. Chinnathambi S. (2018). Multi-faceted role of melatonin in neuroprotection and amelioration of Tau aggregates in Alzheimer’s disease. J. Alzheimer's Dis. 62 (4), 1481–1493. 10.3233/JAD-170900 29562506

[B8] Bar‐Ziv R. Bolas T. Dillin A. (2020). Systemic effects of mitochondrial stress. EMBO Rep. 21 (6), e50094. 10.15252/embr.202050094 32449292 PMC7271648

[B9] Basu A. (2022). The interplay between apoptosis and cellular senescence: bcl-2 family proteins as targets for cancer therapy. Pharmacol. Ther. 230, 107943. 10.1016/j.pharmthera.2021.107943 34182005

[B10] Benz C. C. Yau C. (2008). Ageing, oxidative stress and cancer: paradigms in parallax. Nat. Rev. Cancer 8 (11), 875–879. 10.1038/nrc2522 18948997 PMC2603471

[B11] Bernardini J. P. Lazarou M. Dewson G. (2017). Parkin and mitophagy in cancer. Oncogene 36 (10), 1315–1327. 10.1038/onc.2016.302 27593930

[B12] Birsoy K. Wang T. Chen W. W. Freinkman E. Abu-Remaileh M. Sabatini D. M. (2015). An essential role of the mitochondrial electron transport chain in cell proliferation is to enable aspartate synthesis. Cell. 162 (3), 540–551. 10.1016/j.cell.2015.07.016 26232224 PMC4522279

[B13] Bogenhagen D. Clayton D. A. (1977). Mouse L cell mitochondrial DNA molecules are selected randomly for replication throughout the cell cycle. Cell. 11 (4), 719–727. 10.1016/0092-8674(77)90286-0 560914

[B14] Bogenhagen D. F. (2012). Mitochondrial DNA nucleoid structure. Biochimica Biophysica Acta (BBA)-Gene Regul. Mech. 1819 (9-10), 914–920. 10.1016/j.bbagrm.2011.11.005 22142616

[B15] Boos F. Labbadia J. Herrmann J. M. (2020). How the mitoprotein-induced stress response safeguards the cytosol: a unified view. Trends Cell. Biol. 30 (3), 241–254. 10.1016/j.tcb.2019.12.003 31964548

[B16] Bowling A. C. Beal M. F. (1995). Bioenergetic and oxidative stress in neurodegenerative diseases. Life Sci. 56 (14), 1151–1171. 10.1016/0024-3205(95)00055-b 7475893

[B175] Boya P. (2024). Mitophagy curtails cytosolic mtDNA-dependent activation of cGAS/STING inflammation during aging. Nat. Commun., 15 (1), 830. 38280852 10.1038/s41467-024-45044-1 PMC10821893

[B17] Bratic A. Kauppila T. E. Macao B. Grönke S. Siibak T. Stewart J. B. (2015). Complementation between polymerase-and exonuclease-deficient mitochondrial DNA polymerase mutants in genomically engineered flies. Nat. Commun. 6 (1), 8808. 10.1038/ncomms9808 26554610 PMC4773887

[B18] Bratic I. Trifunovic A. (2010). Mitochondrial energy metabolism and ageing. Biochimica Biophysica Acta (BBA)-Bioenergetics 1797 (6-7), 961–967. 10.1016/j.bbabio.2010.01.004 20064485

[B19] Bravo-San Pedro J. M. Kroemer G. Galluzzi L. (2017). Autophagy and mitophagy in cardiovascular disease. Circulation Res. 120 (11), 1812–1824. 10.1161/CIRCRESAHA.117.311082 28546358

[B20] Bua E. Johnson J. Herbst A. Delong B. McKenzie D. Salamat S. (2006). Mitochondrial DNA–deletion mutations accumulate intracellularly to detrimental levels in aged human skeletal muscle fibers. Am. J. Hum. Genet. 79 (3), 469–480. 10.1086/507132 16909385 PMC1559550

[B21] Burtscher J. Soltany A. Visavadiya N. P. Burtscher M. Millet G. P. Khoramipour K. (2023). Mitochondrial stress and mitokines in aging. Aging Cell. 22 (2), e13770. 10.1111/acel.13770 36642986 PMC9924952

[B22] Byun H. O. Jung H. J. Seo Y. H. Lee Y. K. Hwang S. C. Hwang E. S. (2012). GSK3 inactivation is involved in mitochondrial complex IV defect in transforming growth factor (TGF) β1-induced senescence. Exp. Cell. Res. 318 (15), 1808–1819. 10.1016/j.yexcr.2012.04.012 22652454

[B23] Camell C. D. Yousefzadeh M. J. Zhu Y. Prata L. G. L. Huggins M. A. Pierson M. (2021). Senolytics reduce coronavirus-related mortality in old mice. Science 373 (6552), eabe4832. 10.1126/science.abe4832 34103349 PMC8607935

[B24] Camplejohn R. S. Gilchrist R. Easton D. McKenzie-Edwards E. Barnes D. M. Eccles D. M. (2003). Apoptosis, ageing and cancer susceptibility. Br. J. cancer 88 (4), 487–490. 10.1038/sj.bjc.6600767 12592359 PMC2377171

[B25] Casajus Pelegay E. Puzzo F. Yilmazer A. Cagin U. (2019). Targeting mitochondrial defects to increase longevity in animal models of neurodegenerative diseases. Rev. Biomark. Stud. Metabolic Metabolism-Related Disord. 1134, 89–110. 10.1007/978-3-030-12668-1_5 30919333

[B26] Cedikova M. Pitule P. Kripnerova M. Markova M. Kuncova J. (2016). Multiple roles of mitochondria in aging processes. Physiological Res. 65, S519-S531. 10.33549/physiolres.933538 28006935

[B27] Chapman J. Fielder E. Passos J. F. (2019). Mitochondrial dysfunction and cell senescence: deciphering a complex relationship. FEBS Lett. 593 (13), 1566–1579. 10.1002/1873-3468.13498 31211858

[B28] Chatterjee A. Dutta C. P. (1967). Alkaloids of Piper longum Linn—I: structure and synthesis of piperlongumine and piperlonguminine. Tetrahedron 23 (4), 1769–1781. 10.1016/s0040-4020(01)82575-8 6047519

[B29] Chen G. Kroemer G. Kepp O. (2020). Mitophagy: an emerging role in aging and age-associated diseases. Front. Cell. Dev. Biol. 8, 200. 10.3389/fcell.2020.00200 32274386 PMC7113588

[B30] Chen H. Ruiz P. D. McKimpson W. M. Novikov L. Kitsis R. N. Gamble M. J. (2015). MacroH2A1 and ATM play opposing roles in paracrine senescence and the senescence-associated secretory phenotype. Mol. Cell. 59 (5), 719–731. 10.1016/j.molcel.2015.07.011 26300260 PMC4548812

[B31] Chen H. Vermulst M. Wang Y. E. Chomyn A. Prolla T. A. McCaffery J. M. (2010). Mitochondrial fusion is required for mtDNA stability in skeletal muscle and tolerance of mtDNA mutations. Cell. 141 (2), 280–289. 10.1016/j.cell.2010.02.026 20403324 PMC2876819

[B32] Chistiakov D. A. Sobenin I. A. Revin V. V. Orekhov A. N. Bobryshev Y. V. (2014). Mitochondrial aging and age-related dysfunction of mitochondria. BioMed Res. Int. 2014, 238463. 10.1155/2014/238463 24818134 PMC4003832

[B33] Cho S. J. Pronko A. Yang J. Stout-Delgado H. (2023). Impact of senolytic treatment on gene expression in aged lung. Int. J. Mol. Sci. 24 (8), 7628. 10.3390/ijms24087628 37108795 PMC10145650

[B34] Civitarese A. E. Carling S. Heilbronn L. K. Hulver M. H. Ukropcova B. Deutsch W. A. (2007). Calorie restriction increases muscle mitochondrial biogenesis in healthy humans. PLoS Med. 4 (3), e76. 10.1371/journal.pmed.0040076 17341128 PMC1808482

[B35] Cloonan S. M. Kim K. Esteves P. Trian T. Barnes P. J. (2020). Mitochondrial dysfunction in lung ageing and disease. Eur. Respir. Rev. 29 (157), 200165. 10.1183/16000617.0165-2020 33060165 PMC9488551

[B36] Cohen A. A. (2018). Aging across the tree of life: the importance of a comparative perspective for the use of animal models in aging. Biochimica Biophysica Acta (BBA)-Molecular Basis Dis. 1864 (9), 2680–2689. 10.1016/j.bbadis.2017.05.028 28690188

[B37] Conte M. Ostan R. Fabbri C. Santoro A. Guidarelli G. Vitale G. (2019). Human aging and longevity are characterized by high levels of mitokines. Journals Gerontology Ser. A 74 (5), 600–607. 10.1093/gerona/gly153 29955888

[B38] Costello M. J. Brennan L. A. Basu S. Chauss D. Mohamed A. Gilliland K. O. (2013). Autophagy and mitophagy participate in ocular lens organelle degradation. Exp. eye Res. 116, 141–150. 10.1016/j.exer.2013.08.017 24012988 PMC3856666

[B39] Crane J. D. Devries M. C. Safdar A. Hamadeh M. J. Tarnopolsky M. A. (2010). The effect of aging on human skeletal muscle mitochondrial and intramyocellular lipid ultrastructure. Journals Gerontology Ser. A Biomed. Sci. Med. Sci. 65 (2), 119–128. 10.1093/gerona/glp179 19959566

[B40] Cui H. Kong Y. Zhang H. (2012). Oxidative stress, mitochondrial dysfunction, and aging. J. signal Transduct. 2012, 646354. 10.1155/2012/646354 21977319 PMC3184498

[B41] Davalli P. Marverti G. Lauriola A. D’Arca D. (2018). Targeting oxidatively induced DNA damage response in cancer: opportunities for novel cancer therapies. Oxidative Med. Cell. Longev. 2018, 2389523. 10.1155/2018/2389523 PMC5892224 29770165

[B42] Decout A. Katz J. D. Venkatraman S. Ablasser A. (2021). The cGAS–STING pathway as a therapeutic target in inflammatory diseases. Nat. Rev. Immunol. 21 (9), 548–569. 10.1038/s41577-021-00524-z 33833439 PMC8029610

[B43] Ding K. Wang H. Xu J. Li T. Zhang L. Ding Y. (2014). Melatonin stimulates antioxidant enzymes and reduces oxidative stress in experimental traumatic brain injury: the Nrf2–ARE signaling pathway as a potential mechanism. Free Radic. Biol. Med. 73, 1–11. 10.1016/j.freeradbiomed.2014.04.031 24810171

[B44] Dziechciaz M. Filip R. (2014). Biological psychological and social determinants of old age: bio-psycho-social aspects of human aging. Ann. Agric. Environ. Med. 21 (4), 835–838. 10.5604/12321966.1129943 25528930

[B45] Eberle J. Hossini A. M. (2008). Expression and function of bcl-2 proteins in melanoma. Curr. genomics 9 (6), 409–419. 10.2174/138920208785699571 19506730 PMC2691663

[B46] Edgar D. Trifunovic A. (2009). The mtDNA mutator mouse: dissecting mitochondrial involvement in aging. Aging (Albany NY) 1 (12), 1028–1032. 10.18632/aging.100109 20157586 PMC2815752

[B174] Elmore S. (2007). Apoptosis: a review of programmed cell death. Toxicol. Pathol. 35 (4), 495–516. 17562483 10.1080/01926230701320337 PMC2117903

[B47] Evangelou K. Vasileiou P. V. Papaspyropoulos A. Hazapis O. Petty R. Demaria M. (2023). Cellular senescence and cardiovascular diseases: moving to the “heart” of the problem. Physiol. Rev. 103 (1), 609–647. 10.1152/physrev.00007.2022 36049114

[B48] Fivenson E. M. Lautrup S. Sun N. Scheibye-Knudsen M. Stevnsner T. Nilsen H. (2017). Mitophagy in neurodegeneration and aging. Neurochem. Int. 109, 202–209. 10.1016/j.neuint.2017.02.007 28235551 PMC5565781

[B49] Ganley I. G. Simonsen A. (2022). Diversity of mitophagy pathways at a glance. J. Cell. Sci. 135 (23), jcs259748. 10.1242/jcs.259748 36504076 PMC10656428

[B50] Gioscia-Ryan R. A. Battson M. L. Cuevas L. M. Zigler M. C. Sindler A. L. Seals D. R. (2016). Voluntary aerobic exercise increases arterial resilience and mitochondrial health with aging in mice. Aging (Albany NY) 8 (11), 2897–2914. 10.18632/aging.101099 27875805 PMC5191877

[B51] Greaves L. C. Nooteboom M. Elson J. L. Tuppen H. A. Taylor G. A. Commane D. M. (2014). Clonal expansion of early to mid-life mitochondrial DNA point mutations drives mitochondrial dysfunction during human ageing. PLoS Genet. 10 (9), e1004620. 10.1371/journal.pgen.1004620 25232829 PMC4169240

[B52] Greenhalgh D. G. (1998). The role of apoptosis in wound healing. Int. J. Biochem. Cell. Biol. 30 (9), 1019–1030. 10.1016/s1357-2725(98)00058-2 9785465

[B53] Hoshino A. Mita Y. Okawa Y. Ariyoshi M. Iwai-Kanai E. Ueyama T. (2013). Cytosolic p53 inhibits Parkin-mediated mitophagy and promotes mitochondrial dysfunction in the mouse heart. Nat. Commun. 4 (1), 2308. 10.1038/ncomms3308 23917356

[B54] Indo H. P. Yen H. C. Nakanishi I. Matsumoto K. I. Tamura M. Nagano Y. (2015). A mitochondrial superoxide theory for oxidative stress diseases and aging. J. Clin. Biochem. Nutr. 56 (1), 1–7. 10.3164/jcbn.14-42 25834301 PMC4306659

[B55] Iwashita M. Oka N. Ohkubo S. Saito M. Nakahata N. (2007). Piperlongumine, a constituent of Piper longum L., inhibits rabbit platelet aggregation as a thromboxane A2 receptor antagonist. Eur. J. Pharmacol. 570 (1-3), 38–42. 10.1016/j.ejphar.2007.05.073 17618620

[B56] Jang Y. Y. Sharkis S. J. (2007). A low level of reactive oxygen species selects for primitive hematopoietic stem cells that may reside in the low-oxygenic niche. Blood, J. Am. Soc. Hematol. 110 (8), 3056–3063. 10.1182/blood-2007-05-087759 PMC2018677 17595331

[B57] Jiang Y. H. Jiang L. Y. Wang Y. C. Ma D. F. Li X. (2020). Quercetin attenuates atherosclerosis via modulating oxidized LDL-induced endothelial cellular senescence. Front. Pharmacol. 11, 512. 10.3389/fphar.2020.00512 32410992 PMC7198817

[B58] Jiménez-Loygorri J. I. Villarejo-Zori B. Viedma-Poyatos Á. Zapata-Muñoz J. Benítez-Fernández R. Frutos-Lisón M. D. (2024). Mitophagy curtails cytosolic mtDNA-dependent activation of cGAS/STING inflammation during aging. Nat. Commun. 15 (1), 830. 10.1038/s41467-024-45044-1 38280852 PMC10821893

[B59] Jones R. G. Plas D. R. Kubek S. Buzzai M. Mu J. Xu Y. (2005). AMP-activated protein kinase induces a p53-dependent metabolic checkpoint. Mol. Cell. 18 (3), 283–293. 10.1016/j.molcel.2005.03.027 15866171

[B60] Kauppila T. E. Kauppila J. H. Larsson N. G. (2017). Mammalian mitochondria and aging: an update. Cell. metab. 25 (1), 57–71. 10.1016/j.cmet.2016.09.017 28094012

[B61] Kennedy S. R. Salk J. J. Schmitt M. W. Loeb L. A. (2013). Ultra-sensitive sequencing reveals an age-related increase in somatic mitochondrial mutations that are inconsistent with oxidative damage. PLoS Genet. 9 (9), e1003794. 10.1371/journal.pgen.1003794 24086148 PMC3784509

[B62] Khan N. Syed D. N. Ahmad N. Mukhtar H. (2013). Fisetin: a dietary antioxidant for health promotion. Antioxidants redox Signal. 19 (2), 151–162. 10.1089/ars.2012.4901 PMC3689181 23121441

[B63] Kietzmann T. Petry A. Shvetsova A. Gerhold J. M. Görlach A. (2017). The epigenetic landscape related to reactive oxygen species formation in the cardiovascular system. Br. J. Pharmacol. 174 (12), 1533–1554. 10.1111/bph.13792 28332701 PMC5446579

[B64] Kizhakekuttu T. J. Widlansky M. E. (2010). Natural antioxidants and hypertension: promise and challenges. Cardiovasc. Ther., 28 (4), e20–e32. 20370791 10.1111/j.1755-5922.2010.00137.x PMC2905473

[B65] Kopustinskiene D. M. Bernatoniene J. (2021). Molecular mechanisms of melatonin-mediated cell protection and signaling in health and disease. Pharmaceutics 13 (2), 129. 10.3390/pharmaceutics13020129 33498316 PMC7909293

[B66] Koshiba T. Detmer S. A. Kaiser J. T. Chen H. McCaffery J. M. Chan D. C. (2004). Structural basis of mitochondrial tethering by mitofusin complexes. Science 305 (5685), 858–862. 10.1126/science.1099793 15297672

[B67] Kowaltowski A. J. (2000). Alternative mitochondrial functions in cell physiopathology: beyond ATP production. Braz. J. Med. Biol. Res. 33, 241–250. 10.1590/s0100-879x2000000200014 10657067

[B68] Kujoth G. C. Bradshaw P. C. Haroon S. Prolla T. A. (2007). The role of mitochondrial DNA mutations in mammalian aging. PLoS Genet. 3 (2), e24. 10.1371/journal.pgen.0030024 17319745 PMC1802824

[B69] Kurosaka K. Takahashi M. Watanabe N. Kobayashi Y. (2003). Silent cleanup of very early apoptotic cells by macrophages. J. Immunol. 171 (9), 4672–4679. 10.4049/jimmunol.171.9.4672 14568942

[B70] Kwak S. Ku S. K. Bae J. S. (2014). Fisetin inhibits high-glucose-induced vascular inflammation *in vitro* and *in vivo* . Inflamm. Res. 63, 779–787. 10.1007/s00011-014-0750-4 24923846

[B71] Lane N. Martin W. (2010). The energetics of genome complexity. Nature 467 (7318), 929–934. 10.1038/nature09486 20962839

[B72] Lee C. Zeng J. Drew B. G. Sallam T. Martin-Montalvo A. Wan J. (2015). The mitochondrial-derived peptide MOTS-c promotes metabolic homeostasis and reduces obesity and insulin resistance. Cell. metab. 21 (3), 443–454. 10.1016/j.cmet.2015.02.009 25738459 PMC4350682

[B73] Lee S. Jeong S. Y. Lim W. C. Kim S. Park Y. Y. Sun X. (2007). Mitochondrial fission and fusion mediators, hFis1 and OPA1, modulate cellular senescence. J. Biol. Chem. 282 (31), 22977–22983. 10.1074/jbc.M700679200 17545159

[B74] Lee S. M. Dho S. H. Ju S. K. Maeng J. S. Kim J. Y. Kwon K. S. (2012). Cytosolic malate dehydrogenase regulates senescence in human fibroblasts. Biogerontology, 13, pp.525–536. 10.1007/s10522-012-9397-0 22971926

[B75] Lemasters J. J. (2005). Dying a thousand deaths: redundant pathways from different organelles to apoptosis and necrosis. Gastroenterology, 129 (1). 10.1053/j.gastro.2005.06.006 16012960

[B76] Levine B. Kroemer G. (2019). Biological functions of autophagy genes: a disease perspective. Cell. 176 (1), 11–42. 10.1016/j.cell.2018.09.048 30633901 PMC6347410

[B77] Li Z. Zhang Z. Ren Y. Wang Y. Fang J. Yue H. (2021). Aging and age‐related diseases: from mechanisms to therapeutic strategies. Biogerontology 22 (2), 165–187. 10.1007/s10522-021-09910-5 33502634 PMC7838467

[B78] Liao Z. Yeo H. L. Wong S. W. Zhao Y. (2021). Cellular senescence: mechanisms and therapeutic potential. Biomedicines 9 (12), 1769. 10.3390/biomedicines9121769 34944585 PMC8698401

[B79] Liu J. P. (2014). Molecular mechanisms of ageing and related diseases. Clin. Exp. Pharmacol. Physiology 41 (7), 445–458. 10.1111/1440-1681.12247 24798238

[B80] Liu L. Trimarchi J. R. Smith P. J. Keefe D. L. (2002). Mitochondrial dysfunction leads to telomere attrition and genomic instability. Aging Cell. 1 (1), 40–46. 10.1046/j.1474-9728.2002.00004.x 12882352

[B81] Liu Y. Fiskum G. Schubert D. (2002). Generation of reactive oxygen species by the mitochondrial electron transport chain. J. Neurochem. 80 (5), 780–787. 10.1046/j.0022-3042.2002.00744.x 11948241

[B82] López-Otín C. Blasco M. A. Partridge L. Serrano M. Kroemer G. (2013). The hallmarks of aging. Cell. 153, 1194–1217. 10.1016/j.cell.2013.05.039 23746838 PMC3836174

[B83] Madeo F. Tavernarakis N. Kroemer G. (2010). Can autophagy promote longevity? Nat. Cell. Biol. 12 (9), 842–846. 10.1038/ncb0910-842 20811357

[B84] Madreiter-Sokolowski C. T. Sokolowski A. A. Waldeck-Weiermair M. Malli R. Graier W. F. (2018). Targeting mitochondria to counteract age-related cellular dysfunction. Genes. 9 (3), 165. 10.3390/genes9030165 29547561 PMC5867886

[B85] Mahmoud Y. I. Hegazy H. G. (2016). Ginger and alpha lipoic acid ameliorate age-related ultrastructural changes in rat liver. Biotech. Histochem. 91 (2), 86–95. 10.3109/10520295.2015.1076578 26528730

[B86] Manczak M. Calkins M. J. Reddy P. H. (2011). Impaired mitochondrial dynamics and abnormal interaction of amyloid beta with mitochondrial protein Drp1 in neurons from patients with Alzheimer's disease: implications for neuronal damage. Hum. Mol. Genet. 20 (13), 2495–2509. 10.1093/hmg/ddr139 21459773 PMC3109997

[B87] Marković Z. S. Dimitrić Marković J. M. Milenković D. Filipović N. (2011). Mechanistic study of the structure–activity relationship for the free radical scavenging activity of baicalein. J. Mol. Model. 17, 2575–2584. 10.1007/s00894-010-0942-y 21229369

[B88] Martini H. Passos J. F. (2023). Cellular senescence: all roads lead to mitochondria. FEBS J. 290 (5), 1186–1202. 10.1111/febs.16361 35048548 PMC9296701

[B89] Marycz K. Kornicka K. Basinska K. Czyrek A. (2016). Equine metabolic syndrome affects viability, senescence, and stress factors of equine adipose-derived mesenchymal stromal stem cells: new insight into EqASCs isolated from EMS horses in the context of their aging. Oxidative Med. Cell. Longev. 2016, 1–17. 10.1155/2016/4710326 PMC4670679 26682006

[B90] Marzetti E. Leeuwenburgh C. (2006). Skeletal muscle apoptosis, sarcopenia and frailty at old age. Exp. Gerontol. 41 (12), 1234–1238. 10.1016/j.exger.2006.08.011 17052879

[B91] Mitchell S. J. Scheibye-Knudsen M. Longo D. L. de Cabo R. (2015). Animal models of aging research: implications for human aging and age-related diseases. Annu. Rev. Anim. Biosci. 3 (1), 283–303. 10.1146/annurev-animal-022114-110829 25689319

[B92] Moiseeva O. Bourdeau V. Roux A. Deschênes-Simard X. Ferbeyre G. (2009). Mitochondrial dysfunction contributes to oncogene-induced senescence. Mol. Cell. Biol. 29 (16), 4495–4507. 10.1128/MCB.01868-08 19528227 PMC2725737

[B93] Molenaars M. Janssens G. E. Williams E. G. Jongejan A. Lan J. Rabot S. (2020). A conserved mito-cytosolic translational balance links two longevity pathways. Cell. metab. 31 (3), 549–563. 10.1016/j.cmet.2020.01.011 32084377 PMC7214782

[B94] Mondello C. Scovassi A. I. (2010). Apoptosis: a way to maintain healthy individuals. Genome Stab. Hum. Dis. 50, 307–323. 10.1007/978-90-481-3471-7_16 20012589

[B95] Morita M. Gravel S. P. Chenard V. Sikström K. Zheng L. Alain T. (2013). mTORC1 controls mitochondrial activity and biogenesis through 4E-BP-dependent translational regulation. Cell. metab. 18 (5), 698–711. 10.1016/j.cmet.2013.10.001 24206664

[B96] Morita M. Gravel S. P. Hulea L. Larsson O. Pollak M. St-Pierre J. (2015). mTOR coordinates protein synthesis, mitochondrial activity and proliferation. Cell. cycle 14 (4), 473–480. 10.4161/15384101.2014.991572 25590164 PMC4615141

[B97] Morita M. Prudent J. Basu K. Goyon V. Katsumura S. Hulea L. (2017). mTOR controls mitochondrial dynamics and cell survival via MTFP1. Mol. Cell. 67 (6), 922–935. 10.1016/j.molcel.2017.08.013 28918902

[B98] Mortensen M. Ferguson D. J. Simon A. K. (2010). Mitochondrial clearance by autophagy in developing erythrocytes: clearly important, but just how much so? Cell. cycle 9 (10), 1901–1906. 10.4161/cc.9.10.11603 20495377

[B99] Mottis A. Herzig S. Auwerx J. (2019). Mitocellular communication: shaping health and disease. Science 366 (6467), 827–832. 10.1126/science.aax3768 31727828

[B100] Nehlin J. O. (2023). Senolytic and senomorphic interventions to defy senescence-associated mitochondrial dysfunction. Adv. protein Chem. Struct. Biol. 136, 217–247. 10.1016/bs.apcsb.2023.02.020 37437979

[B101] Nicolson G. L. (2014). Mitochondrial dysfunction and chronic disease: treatment with natural supplements. Integr. Med. A Clinician's J. 13 (4), 35–43. PMC4566449 26770107

[B102] Nicolson G. L. Ash M. E. (2017). Membrane Lipid Replacement for chronic illnesses, aging and cancer using oral glycerolphospholipid formulations with fructooligosaccharides to restore phospholipid function in cellular membranes, organelles, cells and tissues. Biochimica Biophysica Acta (BBA)-Biomembranes 1859 (9), 1704–1724. 10.1016/j.bbamem.2017.04.013 28432031

[B103] Nilsson M. I. Tarnopolsky M. A. (2019). Mitochondria and aging—the role of exercise as a countermeasure. Biology 8 (2), 40. 10.3390/biology8020040 31083586 PMC6627948

[B104] Nissanka N. Moraes C. T. (2018). Mitochondrial DNA damage and reactive oxygen species in neurodegenerative disease. FEBS Lett. 592 (5), 728–742. 10.1002/1873-3468.12956 29281123 PMC6942696

[B105] Norat P. Soldozy S. Sokolowski J. D. Gorick C. M. Kumar J. S. Chae Y. (2020). Mitochondrial dysfunction in neurological disorders: exploring mitochondrial transplantation. NPJ Regen. Med. 5 (1), 22. 10.1038/s41536-020-00107-x 33298971 PMC7683736

[B106] Ohsumi Y. (2014). Historical landmarks of autophagy research. Cell. Res. 24 (1), 9–23. 10.1038/cr.2013.169 24366340 PMC3879711

[B107] Palikaras K. Lionaki E. Tavernarakis N. (2018). Mechanisms of mitophagy in cellular homeostasis, physiology and pathology. Nat. Cell. Biol. 20 (9), 1013–1022. 10.1038/s41556-018-0176-2 30154567

[B108] Palomera-Avalos V. Griñán-Ferré C. Puigoriol-Ilamola D. Camins A. Sanfeliu C. Canudas A. M. (2017). Resveratrol protects SAMP8 brain under metabolic stress: focus on mitochondrial function and Wnt pathway. Mol. Neurobiol. 54, 1661–1676. 10.1007/s12035-016-9770-0 26873850

[B109] Papackova Z. Cahova M. (2014). Important role of autophagy in regulation of metabolic processes in health, disease and aging. Physiological Res. 63 (4), 409–420. 10.33549/physiolres.932684 24702497

[B110] Paraskevaidi M. Martin-Hirsch P. L. Kyrgiou M. Martin F. L., 2017. Underlying role of mitochondrial mutagenesis in the pathogenesis of a disease and current approaches for translational research. Mutagenesis, 32(3), pp.347–342. 10.1038/msb.2010.5 27816931

[B111] Passos J. F. Nelson G. Wang C. Richter T. Simillion C. Proctor C. J. (2010). Feedback between p21 and reactive oxygen production is necessary for cell senescence. Mol. Syst. Biol., 6(1), p.347. 10.1038/msb.2010.5 20160708 PMC2835567

[B112] Passos J. F. Saretzki G. Ahmed S. Nelson G. Richter T. Peters H. (2007). Mitochondrial dysfunction accounts for the stochastic heterogeneity in telomere-dependent senescence. PLoS Biol. 5 (5), e110. 10.1371/journal.pbio.0050110 17472436 PMC1858712

[B113] Pérez V. I. Van Remmen H. Bokov A. Epstein C. J. Vijg J. Richardson A. (2009). The overexpression of major antioxidant enzymes does not extend the lifespan of mice. Aging Cell. 8 (1), 73–75. 10.1111/j.1474-9726.2008.00449.x 19077044 PMC2667893

[B114] Pollack M. Leeuwenburgh C. (2001). Apoptosis and aging: role of the mitochondria. Journals Gerontology Ser. A Biol. Sci. Med. Sci. 56 (11), B475–B482. 10.1093/gerona/56.11.b475 11682568

[B115] Price N. L. Gomes A. P. Ling A. J. Duarte F. V. Martin-Montalvo A. North B. J. (2012). SIRT1 is required for AMPK activation and the beneficial effects of resveratrol on mitochondrial function. Cell. metab. 15 (5), 675–690. 10.1016/j.cmet.2012.04.003 22560220 PMC3545644

[B116] Rai S. N. Singh C. Singh A. Singh M. P. Singh B. K. (2020). Mitochondrial dysfunction: a potential therapeutic target to treat Alzheimer’s disease. Mol. Neurobiol. 57, 3075–3088. 10.1007/s12035-020-01945-y 32462551

[B117] Ravindran J. Gupta N. Agrawal M. Bala Bhaskar A. S. Lakshmana Rao P. V. (2011). Modulation of ROS/MAPK signaling pathways by okadaic acid leads to cell death via, mitochondrial mediated caspase-dependent mechanism. Apoptosis 16, 145–161. 10.1007/s10495-010-0554-0 21082355

[B176] Rinnerthaler M. Bischof J. Streubel M. K. Trost A. Richter K. (2015). Oxidative stress in aging human skin. Biomolecules 5 (2), 545–589. 25906193 10.3390/biom5020545 PMC4496685

[B118] Ristow M. Schmeisser K. (2014). Mitohormesis: promoting health and lifespan by increased levels of reactive oxygen species (ROS). Dose-response 12 (2), 288–341. 10.2203/dose-response.13-035.Ristow 24910588 PMC4036400

[B119] Rizwan S. ReddySekhar P. MalikAsrar B. (2014). Reactive oxygen species in inflammation and tissue injury. Antioxidants redox Signal. 20, 1126–1167. 10.1089/ars.2012.5149 PMC3929010 23991888

[B120] Ross J. M. Coppotelli G. Hoffer B. J. Olson L. (2014). Maternally transmitted mitochondrial DNA mutations can reduce lifespan. Sci. Rep. 4 (1), 6569. 10.1038/srep06569 25299268 PMC4190956

[B121] Ross J. M. Stewart J. B. Hagström E. Brené S. Mourier A. Coppotelli G. (2013). Germline mitochondrial DNA mutations aggravate ageing and can impair brain development. Nature 501 (7467), 412–415. 10.1038/nature12474 23965628 PMC3820420

[B122] Roy M. Reddy P. H. Iijima M. Sesaki H. (2015). Mitochondrial division and fusion in metabolism. Curr. Opin. Cell. Biol. 33, 111–118. 10.1016/j.ceb.2015.02.001 25703628 PMC4380865

[B123] Rubinsztein D. C. Mariño G. Kroemer G. (2011). Autophagy and aging. Cell. 146 (5), 682–695. 10.1016/j.cell.2011.07.030 21884931

[B124] Saccon T. D. Nagpal R. Yadav H. Cavalcante M. B. Nunes A. D. D. C. Schneider A. (2021). Senolytic combination of dasatinib and quercetin alleviates intestinal senescence and inflammation and modulates the gut microbiome in aged mice. Journals Gerontology Ser. A 76 (11), 1895–1905. 10.1093/gerona/glab002 PMC8514064 33406219

[B125] Salabei J. K. Hill B. G. (2013). Mitochondrial fission induced by platelet-derived growth factor regulates vascular smooth muscle cell bioenergetics and cell proliferation. Redox Biol. 1 (1), 542–551. 10.1016/j.redox.2013.10.011 24273737 PMC3836280

[B126] Sato M. Sato K. (2013). Maternal inheritance of mitochondrial DNA by diverse mechanisms to eliminate paternal mitochondrial DNA. Biochimica Biophysica Acta (BBA)-Molecular Cell. Res. 1833 (8), 1979–1984. 10.1016/j.bbamcr.2013.03.010 23524114

[B177] Scott A. C. Dündar F. Zumbo P. Chandran S. S. Klebanoff C. A. Shakiba M. (2019). TOX is a critical regulator of tumour-specific T cell differentiation. Nature 571 (7764), 270–274. 31207604 10.1038/s41586-019-1324-y PMC7698992

[B127] Scarpulla R. C. (2008). Nuclear control of respiratory chain expression by nuclear respiratory factors and PGC‐1‐related coactivator. Ann. N. Y. Acad. Sci. 1147 (1), 321–334. 10.1196/annals.1427.006 19076454 PMC2853241

[B128] Sevrioukova I. F. (2011). Apoptosis-inducing factor: structure, function, and redox regulation. Antioxidants redox Signal. 14 (12), 2545–2579. 10.1089/ars.2010.3445 PMC3096518 20868295

[B129] Shao Y. Sun L. Yang G. Wang W. Liu X. Du T. (2022). Icariin protects vertebral endplate chondrocytes against apoptosis and degeneration via activating Nrf-2/HO-1 pathway. Front. Pharmacol. 13, 937502. 10.3389/fphar.2022.937502 36176424 PMC9513224

[B130] Shigenaga M. K. Hagen T. M. Ames B. N. (1994). Oxidative damage and mitochondrial decay in aging. Proc. Natl. Acad. Sci. 91 (23), 10771–10778. 10.1073/pnas.91.23.10771 7971961 PMC45108

[B131] Short K. R. Bigelow M. L. Kahl J. Singh R. Coenen-Schimke J. Raghavakaimal S. (2005). Decline in skeletal muscle mitochondrial function with aging in humans. Proc. Natl. Acad. Sci. 102 (15), 5618–5623. 10.1073/pnas.0501559102 15800038 PMC556267

[B132] Shum L. C. White N. S. Nadtochiy S. M. Bentley K. L. D. M. Brookes P. S. Jonason J. H. (2016). Cyclophilin D knock-out mice show enhanced resistance to osteoporosis and to metabolic changes observed in aging bone. PloS one 11 (5), e0155709. 10.1371/journal.pone.0155709 27183225 PMC4868300

[B133] Silva Ramos E. Motori E. Brüser C. Kühl I. Yeroslaviz A. Ruzzenente B. (2019). Mitochondrial fusion is required for regulation of mitochondrial DNA replication. PLoS Genet. 15 (6), e1008085. 10.1371/journal.pgen.1008085 31170154 PMC6553695

[B134] Sonawane S. K. Balmik A. A. Boral D. Ramasamy S. Chinnathambi S. (2019). Baicalein suppresses Repeat Tau fibrillization by sequestering oligomers. Archives Biochem. biophysics 675, 108119. 10.1016/j.abb.2019.108119 31568753

[B135] Srivastava S. (2017). The mitochondrial basis of aging and age-related disorders. Genes. 8 (12), 398. 10.3390/genes8120398 29257072 PMC5748716

[B136] Stancu A. L. (2015). AMPK activation can delay aging. Discoveries 3 (4), e53. 10.15190/d.2015.45 32309575 PMC6941559

[B137] Sun N. Youle R. J. Finkel T. (2016). The mitochondrial basis of aging. Mol. Cell. 61 (5), 654–666. 10.1016/j.molcel.2016.01.028 26942670 PMC4779179

[B138] Sun N. Yun J. Liu J. Malide D. Liu C. Rovira I. I. (2015). Measuring *in vivo* mitophagy. Mol. Cell. 60 (4), 685–696. 10.1016/j.molcel.2015.10.009 26549682 PMC4656081

[B139] Taanman J. W. (1999). The mitochondrial genome: structure, transcription, translation and replication. Biochimica Biophysica Acta (BBA)-Bioenergetics 1410 (2), 103–123. 10.1016/s0005-2728(98)00161-3 10076021

[B140] Tan D. X. Manchester L. C. Qin L. Reiter R. J. (2016). Melatonin: a mitochondrial targeting molecule involving mitochondrial protection and dynamics. Int. J. Mol. Sci. 17 (12), 2124. 10.3390/ijms17122124 27999288 PMC5187924

[B141] Taniguchi H. Tanisawa K. Sun X. Kubo T. Higuchi M. (2016). Endurance exercise reduces hepatic fat content and serum fibroblast growth factor 21 levels in elderly men. J. Clin. Endocrinol. 101 (1), 191–198. 10.1210/jc.2015-3308 26562755

[B142] Terman A. Kurz T. Navratil M. Arriaga E. A. Brunk U. T. (2010). Mitochondrial turnover and aging of long-lived postmitotic cells: the mitochondrial–lysosomal axis theory of aging. Antioxidants redox Signal. 12 (4), 503–535. 10.1089/ars.2009.2598 PMC2861545 19650712

[B143] Tower J. (2015). Programmed cell death in aging. Ageing Res. Rev. 23, 90–100. 10.1016/j.arr.2015.04.002 25862945 PMC4480161

[B144] Toyama E. Q. Herzig S. Courchet J. Lewis Jr T. L. Losón O. C. Hellberg K. (2016). Metabolism. AMP-activated protein kinase mediates mitochondrial fission in response to energy stress. Science 351 (6270), 275–281. 10.1126/science.aab4138 26816379 PMC4852862

[B145] Trifunovic A. Wredenberg A. Falkenberg M. Spelbrink J. N. Rovio A. T. Bruder C. E. (2004). Premature ageing in mice expressing defective mitochondrial DNA polymerase. Nature 429 (6990), 417–423. 10.1038/nature02517 15164064

[B146] Turk P. W. Laayoun A. Smith S. S. Weitzman S. A. (1995). DNA adduct 8-hydroxyl-2′-deoxyguanosine (8-hydroxyguanine) affects function of human DNA methyltransferase. Carcinogenesis 16 (5), 1253–1255. 10.1093/carcin/16.5.1253 7767994

[B147] Tzameli I. (2012). The evolving role of mitochondria in metabolism. Trends Endocrinol. Metabolism 23 (9), 417–419. 10.1016/j.tem.2012.07.008 22901785

[B148] United Nations Department of Economic and Social Affairs (2020). World population ageing 2020 highlights: living arrangements of older persons. USA: ST/ESA/SER.A/451.

[B149] Van der Rijt S. Molenaars M. McIntyre R. L. Janssens G. E. Houtkooper R. H. (2020). Integrating the hallmarks of aging throughout the tree of life: a focus on mitochondrial dysfunction. Front. Cell. Dev. Biol. 8, 594416. 10.3389/fcell.2020.594416 33324647 PMC7726203

[B150] van der Veer E. Ho C. O'Neil C. Barbosa N. Scott R. Cregan S. P. (2007). Extension of human cell lifespan by nicotinamide phosphoribosyltransferase. J. Biol. Chem. 282 (15), 10841–10845. 10.1074/jbc.C700018200 17307730

[B151] Van Deursen J. M. (2014). The role of senescent cells in ageing. Nature 509 (7501), 439–446. 10.1038/nature13193 24848057 PMC4214092

[B152] Wall C. E. Rose C. M. Adrian M. Zeng Y. J. Kirkpatrick D. S. Bingol B. (2019). PPEF2 opposes PINK1-mediated mitochondrial quality control by dephosphorylating ubiquitin. Cell. Rep. 29 (10), 3280–3292. 10.1016/j.celrep.2019.10.130 31801089

[B153] Wallace D. C. (2010). Mitochondrial DNA mutations in disease and aging. Environ. Mol. Mutagen. 51 (5), 440–450. 10.1002/em.20586 20544884

[B154] Wang J. Toan S. Zhou H. (2020a). New insights into the role of mitochondria in cardiac microvascular ischemia/reperfusion injury. Angiogenesis 23, 299–314. 10.1007/s10456-020-09720-2 32246225

[B155] Wang L. Ma Y. Wei W. Wan P. Liu K. Xu M. (2020b). Cadherin repeat 5 mutation associated with Bt resistance in a field-derived strain of pink bollworm. Sci. Rep. 10 (1), 16840. 10.1038/s41598-020-74102-z 33033325 PMC7544870

[B156] Wang R. Li J. Niu D. B. Xu F. Y. Zeng X. A. (2021). Protective effect of baicalein on DNA oxidative damage and its binding mechanism with DNA: an *in vitro* and molecular docking study. Spectrochimica Acta Part A Mol. Biomol. Spectrosc. 253, 119605. 10.1016/j.saa.2021.119605 33667888

[B157] Wiley C. D. Velarde M. C. Lecot P. Liu S. U. Sarnoski E. A. Freund A. (2016). Mitochondrial dysfunction induces senescence with a distinct secretory phenotype. Cell. metab. 23 (2), 303–314. 10.1016/j.cmet.2015.11.011 26686024 PMC4749409

[B158] Wu Y. Sun L. Zhuang Z. Hu X. Dong D. (2022). Mitochondrial-derived peptides in diabetes and its complications. Front. Endocrinol. 12, 808120. 10.3389/fendo.2021.808120 PMC8851315 35185787

[B159] Xu M. Pirtskhalava T. Farr J. N. Weigand B. M. Palmer A. K. Weivoda M. M. (2018). Senolytics improve physical function and increase lifespan in old age. Nat. Med. 24 (8), 1246–1256. 10.1038/s41591-018-0092-9 29988130 PMC6082705

[B160] Yan L. J. (2014). Positive oxidative stress in aging and aging-related disease tolerance. Redox Biol. 2, 165–169. 10.1016/j.redox.2014.01.002 25460727 PMC4297947

[B161] Yang S. Lian G. (2020). ROS and diseases: role in metabolism and energy supply. Mol. Cell. Biochem. 467, 1–12. 10.1007/s11010-019-03667-9 31813106 PMC7089381

[B162] Yen T. C. Chen Y. S. King K. L. Yeh S. H. Wei Y. H. (1989). Liver mitochondrial respiratory functions decline with age. Biochem. biophysical Res. Commun. 165 (3), 944–1003. 10.1016/0006-291x(89)92701-0 2610701

[B163] Yoon Y. S. Byun H. O. Cho H. Kim B. K. Yoon G. (2003). Complex II defect via down-regulation of iron-sulfur subunit induces mitochondrial dysfunction and cell cycle delay in iron chelation-induced senescence-associated growth arrest. J. Biol. Chem. 278 (51), 51577–51586. 10.1074/jbc.M308489200 14512425

[B164] Yoon Y. S. Yoon D. S. Lim I. K. Yoon S. H. Chung H. Y. Rojo M. (2006). Formation of elongated giant mitochondria in DFO‐induced cellular senescence: involvement of enhanced fusion process through modulation of Fis1. J. Cell. physiology 209 (2), 468–480. 10.1002/jcp.20753 16883569

[B165] Youm Y. H. Horvath T. L. Mangelsdorf D. J. Kliewer S. A. Dixit V. D. (2016). Prolongevity hormone FGF21 protects against immune senescence by delaying age-related thymic involution. Proc. Natl. Acad. Sci. 113 (4), 1026–1031. 10.1073/pnas.1514511113 26755598 PMC4743827

[B166] Yousefzadeh M. J. Zhu Y. I. McGowan S. J. Angelini L. Fuhrmann-Stroissnigg H. Xu M. (2018). Fisetin is a senotherapeutic that extends health and lifespan. EBioMedicine 36, 18–28. 10.1016/j.ebiom.2018.09.015 30279143 PMC6197652

[B167] Zhang H. Fealy C. E. Kirwan J. P. (2019). Exercise training promotes a GDF15-associated reduction in fat mass in older adults with obesity. Am. J. Physiology-Endocrinology Metabolism 316 (5), E829-E836–E836. 10.1152/ajpendo.00439.2018 PMC6580172 30860878

[B168] Zhang H. Menzies K. J. Auwerx J. (2018). The role of mitochondria in stem cell fate and aging. Development 145 (8), dev143420. 10.1242/dev.143420 29654217 PMC5964648

[B169] Zhou Z. Fan Y. Zong R. Tan K. (2022). The mitochondrial unfolded protein response: a multitasking giant in the fight against human diseases. Ageing Res. Rev. 81, 101702. 10.1016/j.arr.2022.101702 35908669

[B170] Zhu J. Wang K. Z. Chu C. T. (2013). After the banquet: mitochondrial biogenesis, mitophagy, and cell survival. Autophagy 9 (11), 1663–1676. 10.4161/auto.24135 23787782 PMC4028332

[B171] Ziegler D. V. Wiley C. D. Velarde M. C. (2015). Mitochondrial effectors of cellular senescence: beyond the free radical theory of aging. Aging Cell. 14 (1), 1–7. 10.1111/acel.12287 25399755 PMC4310776

[B172] Zimmermann A. Madreiter-Sokolowski C. Stryeck S. Abdellatif M. (2021). Targeting the mitochondria-proteostasis axis to delay aging. Front. Cell. Dev. Biol. 9, 656201. 10.3389/fcell.2021.656201 33777963 PMC7991595

[B173] Zorov D. B. Juhaszova M. Sollott S. J. (2014). Mitochondrial reactive oxygen species (ROS) and ROS-induced ROS release. Physiol. Rev. 94 (3), 909–950. 10.1152/physrev.00026.2013 24987008 PMC4101632

